# Antimicrobial Properties of Ti- and Zr-Based Nanotextured Thin Film Metallic Glasses Against *Pseudomonas aeruginosa*

**DOI:** 10.3390/biom16060759

**Published:** 2026-05-22

**Authors:** Chijioke R. Onyeagba, Jonathan M. Harris, Timothy E. Egbo, Cameron Brown, Hongxia Wang, Tuquabo Tesfamichael

**Affiliations:** 1 School of Mechanical, Medical and Process Engineering, Faculty of Engineering, Queensland University of Technology, Brisbane, QLD 4000, Australia; chijioke.onyeagba@qut.edu.au (C.R.O.); cp.brown@qut.edu.au (C.B.); 2 Centre for Biomedical Technologies, Queensland University of Technology, Brisbane, QLD 4000, Australia; 3 Centre for Materials Science, Queensland University of Technology, Brisbane, QLD 4000, Australia; hx.wang@qut.edu.au; 4 School of Biomedical Science, Faculty of Health, Queensland University of Technology, Brisbane, QLD 4000, Australia; j2.harris@qut.edu.au; 5 Walter Reed Army Institute of Research-Armed Forces Research Institute of Medical Sciences, Bangkok 10400, Thailand; 6 School of Chemistry and Physics, Faculty of Science, Queensland University of Technology, Brisbane, QLD 4000, Australia

**Keywords:** metallic glasses, nanotextured thin films, bactericidal efficacy, *P. aeruginosa*, surface wettability, bacterial adhesion, surface colonisation

## Abstract

Nanotextured thin film metallic glasses (TFMGs) have emerged as promising antimicrobial coatings for biomedical applications; however, systematic comparisons across compositionally distinct Ti- and Zr-based systems, as well as their early-stage bactericidal mechanisms, remain limited. Here, we show, for the first time, a comparative, compositionally resolved correlation linking alloy chemistry, nanotexture, and bactericidal mechanisms across polymorphic TFMGs. Three co-sputtered biocompatible coatings (Ti_47_Fe_41_Cu_12_, Zr_71_Fe_3_Al_26_, and Zr_58_W_31_Cu_11_) were deposited on medical-grade titanium and stainless steel (SS316L) via magnetron co-sputtering, producing uniform amorphous films (190–298 nm) with nanoscale roughness of 1.6 ± 0.05 to 8.1 ± 0.05 nm. Surface wettability spanned hydrophilic (71.1 ± 5.6°) to hydrophobic (106.5 ± 3.5°), modulating bacterial interactions. Antimicrobial performance against *Pseudomonas aeruginosa* was evaluated using live/dead fluorescence imaging, quantitative image analysis, and electron microscopy after 2–4 h incubation. All coatings reduced bacterial adhesion and viability relative to bare substrates, with Zr_58_W_31_Cu_11_ achieving >60% reduction in surface-associated bacterial coverage. Time-resolved analysis revealed a rapid transition to predominantly non-viable populations on coated surfaces, in contrast to sustained viability on controls. Mechanistically, bactericidal activity arises from the synergistic coupling of nanotopography-induced membrane stress, wettability-governed adhesion energetics, and in situ formation of CuO, Fe_2_O_3_, WO_3_, and ZrO_2_ oxides that promote electrostatic interactions and proposed reactive oxygen species generation, driving oxidative membrane damage. These results establish a scalable design framework for TFMGs, while highlighting the need for long-term biofilm and electrochemical validation.

## 1. Introduction

The rise of antibiotic-resistant bacteria poses a major challenge in biomedical applications, driving interest in advanced antimicrobial surfaces. Nature-inspired nano-patterned structures (e.g., nanowires, nanopillars, micro-ridges) exhibit bactericidal activity [1,2,3,4,5], but their limited mechanical and chemical durability restricts clinical translation [6,7]. In contrast, nano-morphological surfaces on amorphous matrices offer enhanced strength and stability. Thin film metallic glasses (TFMGs) are therefore proposed as promising antimicrobial coatings due to their high mechanical performance, corrosion resistance, and tunable nanoscale surface reactivity [8,9,10].

Bulk metallic glasses (BMGs), including Zr-, Fe-, and Cu-based systems, exhibit antimicrobial activity relative to SS316L [11,12], but are limited by brittleness and poor surface controllability. In contrast, TFMGs enable controlled nanotextured coatings with reported antibacterial efficacy against *P. aeruginosa*, *S. aureus*, and *E. coli* [13], while transitioning to improved ductility at reduced thickness [14,15].

Metallic glasses are classified as metal–metal or metal–metalloid systems [16,17,18,19], and can incorporate Cu or Ag to enhance antimicrobial performance [16,17,18,19,20]. Ag–Cu systems reduce bacterial colonisation [19,20], while Fe–B, Cu–B, and Ti–B alloys are optimised for strength and wear resistance [18,21]. Aluminium (Al) was incorporated for its lightweight nature and its known contribution to the formation of passive, corrosion-resistant oxide layers. Tungsten (W) was included to enhance the mechanical properties, such as hardness and wear resistance, of the metallic glass coatings. Antimicrobial functionality is further enhanced through alloying with active elements such as Cu or Ag [19,20].

Nanotextured TFMGs can promote metal oxide formation and reactive oxygen species (ROS) generation [22,23], which contribute to bacterial membrane damage and cellular stress [24,25]. Nanoparticle-based systems further inhibit adhesion, colonisation, and biofilm formation, with polymorphic behaviour enabling multi-target antibacterial action [4,26,27].

Previous studies have shown that Zr- and Ti-based metallic glass coatings improve hardness, corrosion resistance, and wear behaviour on Ti and SS316L substrates [8,9,10]. Among them, Zr_58_W_31_Cu_11_, Zr_71_Fe_3_Al_26_, and Ti_47_Fe_41_Cu_12_ exhibit superior mechanical and tribological properties, with Zr_58_W_31_Cu_11_ showing the highest stability. Cu incorporation enhances antimicrobial activity but requires controlled concentration to avoid cytotoxicity.

However, the combined influence of nanotopography, wettability, and ROS-mediated effects on bacterial behaviour across different substrates remains unclear, particularly against clinically relevant biofilm-forming pathogens such as *Pseudomonas aeruginosa* [28,29,30,31,32]. Therefore, this study investigates Ti_47_Fe_41_Cu_12_, Zr_71_Fe_3_Al_26_, and Zr_58_W_31_Cu_11_ TFMG coatings [33,34] on Ti and SS316L substrates to establish structure–property–performance relationships governing antibacterial activity.

## 2. Experimental Methods

### 2.1. Sample Preparation

Ti_47_Fe_41_Cu_12_, Zr_71_Fe_3_Al_26_, and Zr_58_W_31_Cu_11_ coatings were prepared on medical-grade titanium and stainless steel 316L substrates by co-sputtering of high-purity biocompatible elemental targets (99.95–99.99 wt.%). Structural characterisation of the samples was performed using X-ray diffraction (XRD) on a D8 Advance powder diffractometer and a D8 Venture single-crystal system (Bruker, Karlsruhe, Germany). Surface chemical composition was analysed using X-ray photoelectron spectroscopy (XPS) with an Axis Supra system (Kratos Analytical, Manchester, UK). Surface topography and step-height (thickness and surface roughness Ra) measurements were obtained using a Dektak XT-A stylus profilometer (Bruker, Karlsruhe, Germany). Nanoscale surface morphology and roughness were further examined using atomic force microscopy (AFM), employing a Dimension Icon system (Bruker, Karlsruhe, Germany) and a Multiscan Lab ultra-high vacuum variable-temperature AFM (Omicron NanoTechnology, Taunusstein, Germany). Additional AFM measurements were conducted using a Solver Pro system (NT-MDT, Zelenograd, Russia). All instrumentation was accessed through the Central Analytical Research Facility at Queensland University of Technology (QUT), Australia. All measurements are summarised in Table 1 and Table 2.

The co-sputtering process and film characterisation were previously published [33,34] and briefly described in the supplemental document. Supplementary Materials Tables S1–S3 further summarise the mechanical, electrochemical, and compositional characteristics of the coatings, confirming the amorphous nature, corrosion resistance, and stability of the deposited films. Supplementary Materials Figures S3 and S4 additionally support the uniformity and nanoscale morphology of the coatings across the substrates.

The co-sputtering deposition parameters for the Ti- and Zr-based thin film metallic glass (TFMG) coatings are summarised in Table 1. Three ternary systems Ti-Fe-Cu, Zr-Fe-Al, and Zr-W-Cu, were fabricated using controlled sputtering powers assigned to each elemental target. In all cases, the primary matrix-forming element (Ti or Zr) was deposited at a higher power (150 W), while the secondary alloying elements were maintained at 50 W, ensuring consistent compositional tuning across samples. A constant deposition duration of 30 min was employed for all coatings to maintain uniform processing conditions and enable direct comparison of their structural and antimicrobial performance, as investigated in this study.

The polymorphic properties of the as-deposited samples were obtained by XPS, XRD, SEM and AFM and shown in Figure S1 of the Supplementary Materials, and previously described by C.R. Onyeagba et al., 2023 [33]. The thickness of each film (Table 2) as measured by the DektakXT-Bruker stylus profilometer is a function of the individual sputtering yield of the elements. AFM was used to analyse the surface roughness/nanotexture (Table 2 and Figure S1) and affirm the uniformity of the thickness across the substrate. The characteristics of the films, including their mechanical and corrosion resistance properties via nanoindentation and electrochemical polarisation, respectively, were reported in the Supplementary Materials (Tables S1 and S2, Figures S1 and S2) as previously published [33,34]. The surface wettability, morphology, bactericidal properties and efficacy of the samples were examined in detail as described in Section 2.2. and Section 2.3.

### 2.2. Surface Wettability Test

Static contact angles were measured using the sessile drop method (OneAttension, Biolin Scientific, Gothenburg, Sweden) with a droplet volume of 5 μL at ambient conditions. For each sample, measurements were performed at a minimum of five independent locations to ensure statistical reliability. Contact angles were obtained by fitting the droplet profile using the Young–Laplace method.

Water contact angles (WCAs) were measured on each surface using sessile drop analysis. At least five independent measurements were performed per sample to ensure reproducibility. Before wettability and biological testing, samples were sterilised under ultraviolet irradiation (λ = 254 nm) for 30 min per side. UV sterilisation was selected as a non-contact method that avoids chemical contamination. At the applied wavelength and exposure duration, no measurable changes in surface morphology or wettability were observed, consistent with the expected limited interaction of UV-C radiation with metallic and oxide surfaces.

### 2.3. Bactericidal Tests

**Figure 1:**
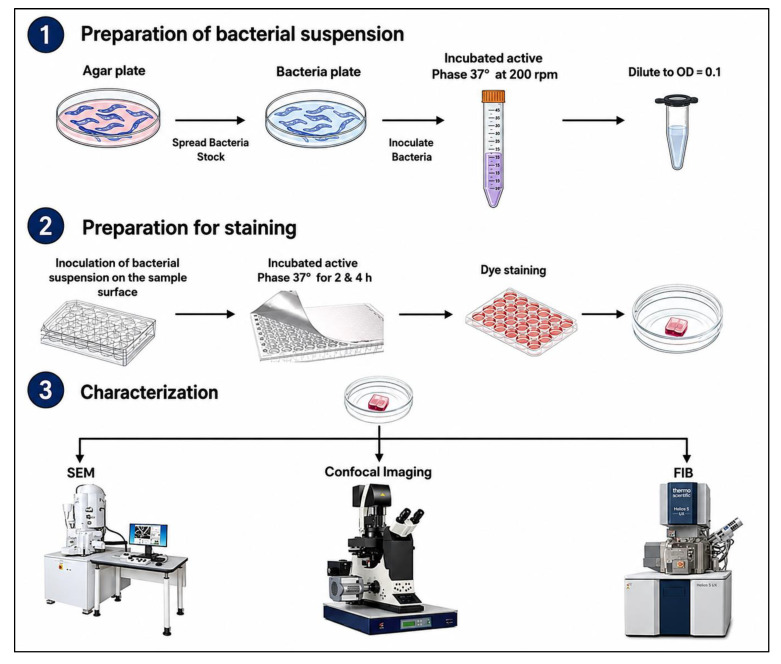
Schematic illustration of the experimental workflow for bacterial suspension preparation, sample staining, and surface characterisation. (1) Bacterial suspension was prepared by culturing bacteria from agar plates, incubating at 37 °C with shaking (200 rpm), and diluting to an optical density (OD) of 0.1. (2) Samples were inoculated with bacterial suspension, incubated for 2 and 4 h, followed by dye staining. (3) Characterisation of bacterial adhesion and surface interactions was performed using scanning electron microscopy (SEM), confocal laser scanning microscopy (CLSM), and focused ion beam (FIB) analysis.

Coated and uncoated samples were sterilised by immersion in 100% ethanol, followed by overnight UV exposure. Sterility was confirmed by incubating sterilised controls without bacterial inoculation. *P. aeruginosa* (ATCC 27853) was cultured in Luria broth and adjusted to OD600 = 0.1. Samples were inoculated and incubated at 37 °C for 2 h and 4 h (see Figure 1, step 1), consistent with prior reports of rapid early surface attachment [35]. While short incubation times provided insight into initial adhesion, extended time points (≥24 h) are recommended in future studies to assess biofilm formation.

After incubation, samples were rinsed with sterile 0.8% NaCl to remove unattached cells. Confocal laser scanning microscopy (CLSM, Leica Microsystems TCS-SP5, Wetzlar, Germany) with SYTO 9/PI staining was used for live/dead imaging. A minimum of six fields of view (FOVs) from three biological replicates were analysed using Fiji (ImageJ 1.54f). Quantitative analysis included colony area, circularity, and live/dead ratios. SEM and Focused Ion Beam (FIB) imaging were performed on dehydrated samples to examine bacterial morphology and surface interactions.

### 2.4. Quantitative Analysis

Bactericidal efficacy (BE) was evaluated both qualitatively (CLSM, SEM, FIB) and quantitatively using live/dead cell ratios. Data are presented as mean ± standard deviation, derived from multiple fields of view and independent replicates. The consistency of trends across replicates was used to support comparative evaluation between samples. The percentage of viable cells was calculated as

(1) $$\mathrm{Active}\mathrm{cell}\mathrm{ratio}\left(\%\right)=\frac{\mathrm{Number}\mathrm{of}\mathrm{green}\mathrm{stained}\mathrm{cells}}{\mathrm{Total}\mathrm{adhered}\mathrm{cells}}\times 100$$

This metric provided comparative insights into bacterial survival across different coatings. For the bacterial viability test, the samples were placed on a thin glass coverslip, facing down (Figure 1 Step 2), and imaged using a Leica_TCSSP5 Confocal Laser Scanning Microscope (CLSM) with filter sets at 63× and 512 × 512 image sizes.

After CLSM imaging, the tested samples were washed gently with 10 mM PBS, fixed with 2.5% glutaraldehyde in cacodylate buffer for 1 h, and dehydrated with a series of ethanol (10 min each in 20, 30, 40, 50, 60, 70, 90, 95, and 100% ethanol). Samples were treated with hexamethyldisilane and allowed to dry overnight in the fume hood, thereby preparing them for Field Emission Scanning Electron Microscopy (FE-SEM) and FIB imaging (Figure 1, step 3). FE-SEM (JEOL JSM-7001F), manufactured by JEOL Ltd., Tokyo, Japan, with an acceleration voltage of 5 kV, was used to investigate the structure and morphology of the attached bacterial cells. FIB was aligned and used to take high-resolution SEM images of the bacterial cells to understand the cell/surface interaction and their mortality as a function of the surface coating.

**Figure 2:**
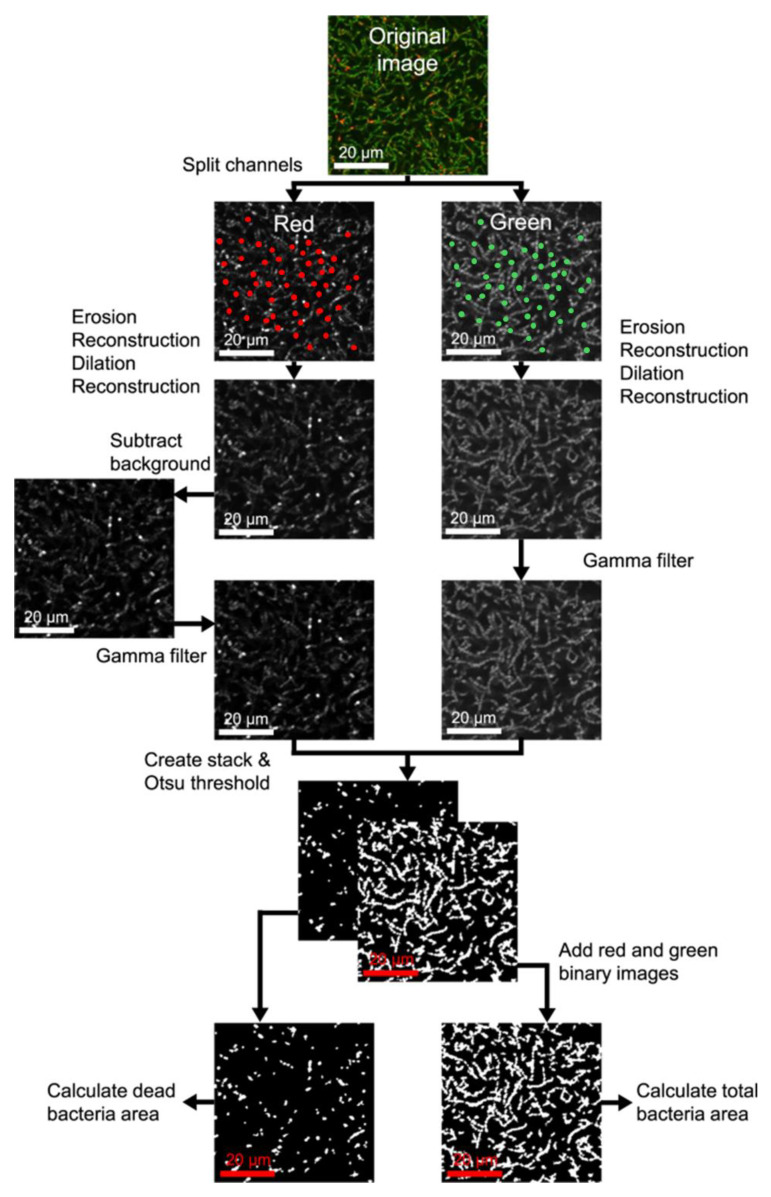
Image analysis workflow using Fiji software, including pre-processing (grayscale conversion, noise reduction, and intensity normalisation) and automated thresholding to segment and quantify regions of interest for accurate and reproducible data analysis (ImageJ, US National Institutes of Health, Bethesda, MD, USA). Reprinted from reference [36]. Copyright © 2021. Sophie E. Mountcastle et al.

The image analysis was performed using Fiji (ImageJ) 1.54f; US National Institutes of Health, Bethesda, MD, USA), as shown in Figure 2. Multiple fields of view (FOVs) were obtained. A minimum of 6 high-quality FOVs of at least 3 replicas were Z-stacked, converted to grayscale (8-bit) and adjusted for optimal brightness/contrast. A threshold was set to distinguish bacterial colonies from the background and to differentiate between live (green) and dead (red) cells. To deduce and analyse the colony counts, size and circularity parameters were set as 10-Infinity pixels^2^ and 0.50–1.00, respectively. The mean count, standard deviations, and area were obtained. The accuracy of counts was verified by manually checking a subset of images. Re-runs were carried out to ensure consistency and accuracy. The analysis of results, summary of statistics, and experimental data plotting were performed using Excel spreadsheet software.

## 3. Results and Discussion

### 3.1. Surface Wettability Analysis

**Figure 3:**
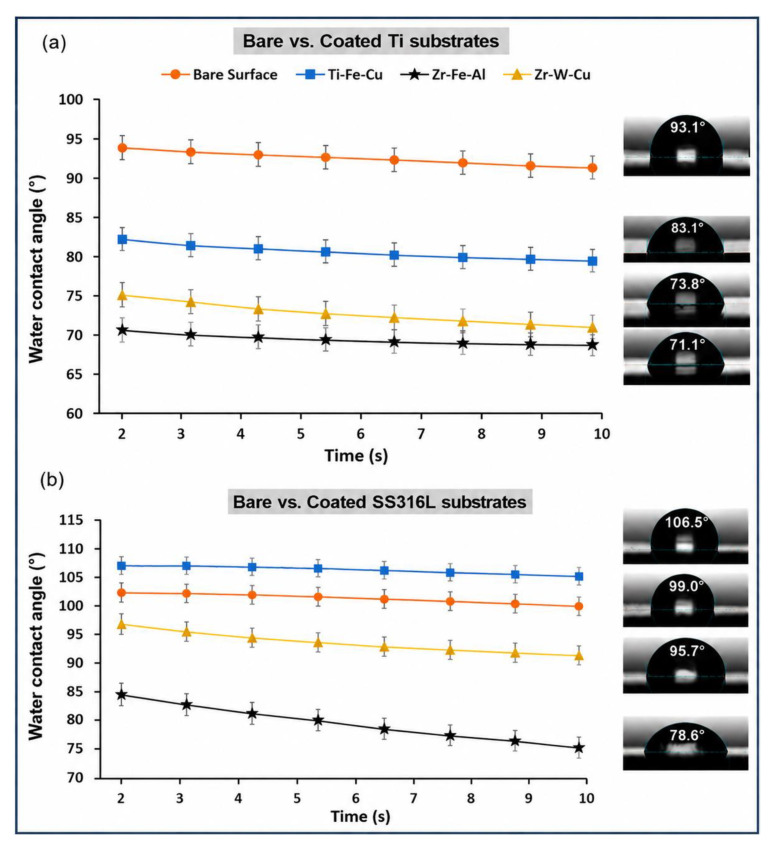
Graphical representation of the water contact angle of the thin films on (**a**) Ti substrate and (**b**) SS316L substrate. For comparison, the water contact angles (WCAs) of the bare substrates are shown. Colored lines and symbols represent different substrate conditions: bare surface (orange circles), Ti-Fe-Cu coating (blue squares), Zr-Fe-Al coating (black stars), and Zr-W-Cu coating (yellow triangles). The arrows indicate the trend of water contact angle variation with time, while the dotted/outlined profile in the droplet images marks the fitted droplet contour used for contact-angle measurement. All values are normalised to the initial measurement at t=2, defined as 100% for each sample, with comparisons made relative to the respective substrate.

Contact angle measurements confirmed that the three coatings on Ti and stainless steel substrates displayed a range of hydrophilic and hydrophobic behaviours, as shown in Table 3 [37]. For example, Zr_71_Fe_3_Al_26_ exhibited hydrophilic behaviour on both substrates, whereas Ti_47_Fe_41_Cu_12_ was hydrophobic on stainless steel substrates but became hydrophilic on the titanium substrate. Variations in wettability correlated with nanoscale roughness measured by AFM, suggesting that both chemistry and topography influenced surface energy. These results are summarised in Table 3 and illustrated in Figure 3.

In simple terms, the thermodynamic mechanism of bacterial adhesion to solid surfaces, as affirmed by the extended Derjaguin–Landau–Verwey–Overbeek (DLVO) theory, suggests that “bacteria with hydrophobic cell membrane prefer hydrophobic material surfaces due to the affinities of bacteria adhesion molecules, such as pili and fimbriae, for the hydrophobic region and those with hydrophilic cell membrane favour hydrophilic material surfaces” [38,39]. However, *P. aeruginosa* has a hydrophobic cell membrane and has been reported to adhere to both hydrophilic and hydrophobic surfaces, as a function of contact time and the bacteria molecules/substrate affinities, respectively [37,40]. *P. aeruginosa* facilitates adhesion through signal-detecting cell development, extracellular polysaccharide (EPS) production, metabolic activity, charge, wettability, cell wall stiffness, and receptor–ligand binding mediated by adhesins [41,42]. Their appendages, such as pili and flagella, also play a role in this process [43,44]. Moreover, their adhesion and the initial formation of biofilm are also influenced by surface charge, wettability, nanotextured surface topography, and chemistry of the substrate [45,46].

In Table 3, all roughness measurements show very low standard deviation, indicating high precision and consistency in the surface roughness data obtained by AFM Whereas the WCA of Zr_71_Fe_3_Al_26_ and Zr_58_W_31_Cu_11_ thin films on SS316L show notable variability, which may reflect surface heterogeneity, droplet instability, or environmental factors during the measurements.

Figure 3 shows the time-dependent wettability of the as-deposited samples compared with that of the bare substrates. The gradual decrease in water contact angle with time is attributed to dynamic wetting effects, including capillary infiltration into the nanotextured surface, surface energy equilibration, and hydration layer formation, resulting in enhanced spreading of the droplet toward a thermodynamically stable state. In Figure 3a, the surfaces of all the films appear to exhibit hydrophilic behaviour despite the hydrophobicity of the bare Ti substrate, indicating positive surface free energies for the films and a negative surface free energy for the bare substrate. However, only the Zr-Fe-Al film on the SS316L substrate shows hydrophilic characteristics with increasing contact time (Figure 3b). The rest of the films and the SS316L substrate have hydrophobic behaviour as shown in Figure 3b. These differences in wettability (hydrophilic vs. hydrophobic) can significantly influence bacterial adhesion and biofilm formation.

Hydrophilic surfaces have been reported to be more conducive to *P. aeruginosa* adhesion due to the formation of hydrogen bonds between bacteria and the hydrophilic surface as contact time increases [47,48]. This means that there could be more attached bacteria on hydrophilic surfaces with time, despite the initial bacteria holdoff [49], as is the case with a thin film metallic glass on a Ti substrate discussed below. The wettability trends are further supported by the supplementary surface analyses (Figures S3 and S4), which demonstrate nanoscale morphological variations and surface homogeneity that contribute to differences in water spreading behaviour and bacterial interaction.

### 3.2. Bacterial Studies

#### 3.2.1. Bacterial Adhesion

**Figure 4:**
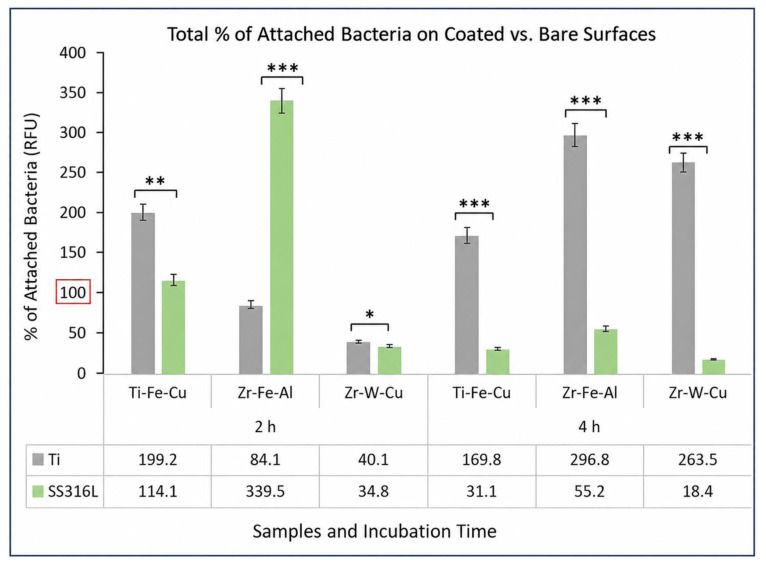
Total percentage of attached bacteria (RFU) on coated (SS316L) vs. bare (Ti) surfaces after 2 h and 4 h of incubation. Data is presented as mean ± SD. Statistical significance between groups was determined using an unpaired *t*-test (*p* < 0.05, *p* < 0.01, *p* < 0.001), where *p* < 0.05 (*), *p* < 0.01 (**), and *p* < 0.001 (***); ns indicates non-significant differences. Values are expressed as a percentage relative to the bare surface condition/adhered population, which was defined as 100%. (highlighted by the red boxed value on the y-axis).

The absence of grain boundaries and long-range order (polymorphous nature of the coatings) can contribute to improved chemical homogeneity, reduced localised corrosion susceptibility, and more uniform surface characteristics [33,34], which may influence bacterial adhesion. The bactericidal efficacy (BE) among several methods can be determined by calculating the percentage reduction in colony-forming units (CFU) from the attached bacteria between a test sample and a control, following ISO standard 22916:2011 [50,51,52] or fluorescence staining, where live and dead fluorescent signals (expressed as relative fluorescent units—RFU) of cells are observed under a microscope after incubation in the dark [53,54]. CFU measures BE for contact-killing surfaces indirectly, as it assumes that all bacterial cells are removed from the substrate and transferred into the suspension. However, it may not accurately capture the number of dead cells and requires precise control of experimental conditions. Moreover, it is effective with antimicrobial drugs and leaching agents in solutions. The RFU method provides a fluorescence-based spatial map rather than absolute bacterial counts and relies on signal intensity interpretation, which may lead to variability associated with the limited field of view. However, this limitation was minimised through standardised image analysis (e.g., ImageJ) and the inclusion of appropriate controls [53], making RFU suitable for this study. After staining, live and dead cells were identified by green (Syto9) and red (PI) signals, respectively. The percentage of adhered cells on thin films relative to bare Ti and SS316L over time is shown in Figure 4, with RFU and wettability used to assess viability and bacterial attachment.

**Figure 5:**
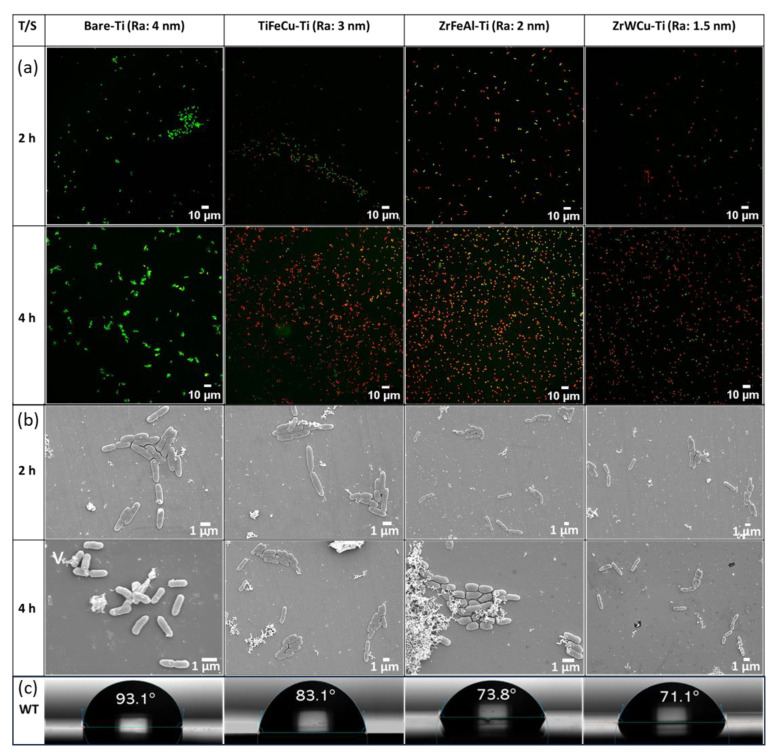
Representative confocal fluorescence images of inoculated bacteria on the sample surfaces showing live/dead staining after incubation: green fluorescence indicates viable/live bacterial cells, while red fluorescence represents non-viable/dead bacterial cells. Yellow/orange regions correspond to overlapping live/dead fluorescence signals. (**a**) Live/dead fluorophore staining images, (**b**) SEM micrographs of *P. aeruginosa* after 2 and 4 h of incubation, and (**c**) wettability properties of bare Ti and the as-deposited thin films reported in Figure 3a. The blue arrow in the water droplet images indicates the contact angle measurement direction/interface used for wettability analysis. Scale bars: 10 µm for confocal images and 1 µm for SEM micrographs.

**Figure 6:**
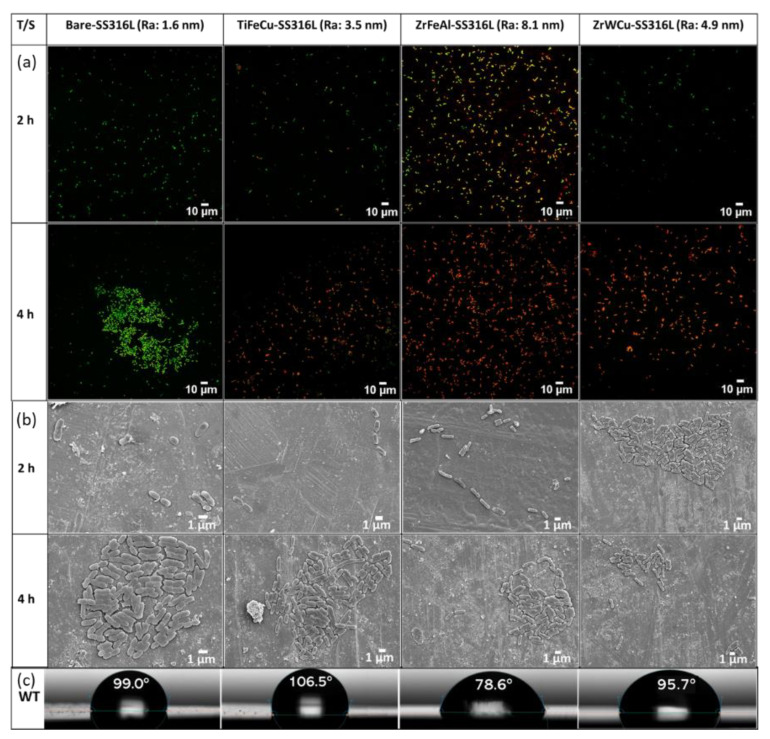
Representative confocal fluorescence images of inoculated bacteria on the sample surfaces showing live/dead staining after incubation: green fluorescence indicates viable/live bacterial cells, while red fluorescence represents non-viable/dead bacterial cells. Yellow/orange regions correspond to overlapping live/dead fluorescence signals. (**a**) Live/dead fluorophore staining images, (**b**) SEM micrographs of *P. aeruginosa* after 2 and 4 h of incubation, and (**c**) wettability properties of bare SS316L and the as-deposited thin films reported in Figure 3b. The blue arrow in the water droplet images indicates the contact angle measurement direction/interface used for wettability analysis. Scale bars: 10 µm for confocal images and 1 µm for SEM micrographs.

**Figure 7:**
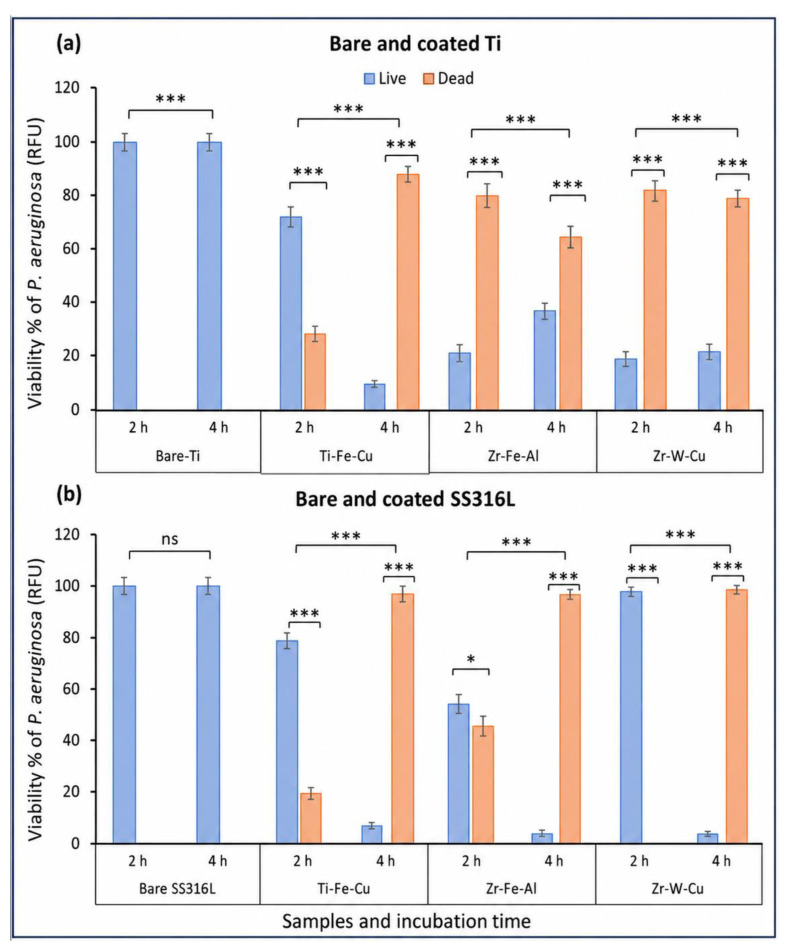
Active cell ratio (%) and corresponding reduction in *Pseudomonas aeruginosa* viability on bare and coated (**a**) Ti and (**b**) SS316L substrates after 2 h and 4 h of incubation. Data are presented as mean ± standard deviation from multiple fields of view and independent replicates. Statistical significance between groups was determined using an unpaired *t*-test, where *p* < 0.05 (*), *p* < 0.01 (**), and *p* < 0.001 (***); ns indicates non-significant differences. Bare substrates show no observable reduction in viability, while coated samples exhibit coating- and time-dependent decreases in active cell ratio.

Figure 4 shows the total population of adhered bacteria colonies on Ti-Fe-Cu, Zr-Fe-Al, and Zr-W-Cu nanotextured metallic glass thin film coatings as a percentage of the total population of attached bacteria on the bare Ti and SS316L control after a 2 h and 4 h incubation period. The total CFU count of the films on SS316L substrate is ≤100% except for the Zr-Fe-Al film, which showed anomality. In contrast, higher CFU values (>100%) were recorded for all the films on the SS316L substrate incubated for a longer period (4 h). The shorter incubation time (2 h) has shown mixed values. As noted above, % bacterial attachment does not reliably quantify cell death; therefore, the figure reflects initial adhesion only, while bactericidal activity is assessed independently using live/dead fluorescence analysis (Figure 5, Figure 6 and Figure 7). Additional quantitative and morphological observations presented in Figures S5 and S6 of the Supplementary Materials further corroborate the reduction in bacterial attachment and altered colony distribution on the coated surfaces compared with the bare substrates. However, it is important to note that the adhesion force on the hydrophilic metallic glass-coated Ti substrate increased after 2 h, as indicated by the higher RFU values in Figure 4. In contrast, the metallic glass-coated SS316L substrates were predominantly hydrophobic (except for the Zr-Fe-Al coating; Figure 3b) and exhibited a progressive reduction in bacterial adhesion over time (Figure 4).

Overall, the early attachment behaviour at 2 h aligns with the thermodynamic expectations described earlier; however, this trend reverses after 4 h. This reversal is attributed to the formation of hydrogen bonds between bacteria and the hydrophilic coatings during prolonged contact [55,56]. This is consistent with the decreasing WCA slope over time for Zr-Fe-Al and Zr-W-Cu in Figure 3, indicating increased surface wetting. Conversely, on hydrophobic substrates, the adhered bacteria gradually lose viability with increasing incubation time, suppressing metabolic activity and, consequently, reducing the number of attached cells. Together, these observations demonstrate that although hydrophobic surfaces initially promote stronger bacterial adhesion compared to hydrophilic surfaces, time-dependent factors, including electrostatic interactions, hydrogen bonding, and bacterial immobilisation, collectively govern the overall adhesion behaviour.

#### 3.2.2. Bacterial Viability of the Thin Films on the Ti Substrate

Figure 5a shows the bacterial viability test of Ti-Fe-Cu, Zr-Fe-Al, and Zr-W-Cu thin films on the Ti substrate as compared with the corresponding bare Ti substrate. From the figure, more viable bacterial cells are shown on the bare Ti surface than on the nanotextured films. Thus, a gradual bacterial community is formed on the bare surface after a 2 h incubation period. The hydrophobic nature of the bare Ti substrate appeared to promote initial bacterial adhesion, and this favours multiple aggregates with possible biofilm formation in the longer incubation period (after 4 h), in contrast to the Ti-Fe-Cu- and Zr-W-Cu-coated Ti substrate with randomly isolated colonies. Moreover, the total RFU on the bare Ti substrate appears viable (green fluorophore) compared to the RFU on the nanotextured films, where there are more dead (red fluorophore) than viable bacteria cells.

Figure 5b shows representative SEM micrographs of adhered rod-shaped *P. aeruginosa* bacteria on coated and bare Ti substrates. The micrographs show fewer attached bacterial cells on the nanotextured Ti-Fe-Cu, Zr-Fe-Al, and Zr-W-Cu thin films, revealing their bactericidal properties compared to the bare substrate. The SEM images show little clusters of bacterial cells on the bare titanium surface with increasing incubation time (4 h), in agreement with the confocal images in Figure 5a. A similar observation has been previously reported by Wu et al. [40], where clusters of *P. aeruginosa* form on micro-rough titanium surfaces with respect to time. The hydrophilic property (Figure 5c) of all the films creates an initial (0–2 h) inhibition of bacteria adhesion to the substrates. In principle, the isolated cell distributions are indicative of the bactericidal efficacy of the thin films, where bacterial cell metabolism is deterred, and may influence early-stage colonisation behaviour due to the presence of nanoparticles and ROS discussed in Section 3.3, “Bactericidal/antibacterial mechanism”. Moreover, multiple bacterial aggregates formed on the Zr-Fe-Al coating after a 4 h incubation period, a different observation from the other two metallic glass films, which showed no clusters. However, these aggregates are composed of dead bacteria, as indicated by the RFU (red fluorophore). It is clear from the above phenomena that the absence of biocidal Cu in Zr-Fe-Al has allowed for the movement of the bacteria and eventually clustering before their death due to the nanotextured surface.

#### 3.2.3. Bacterial Viability of the Thin Films on the SS316L Substrate

The bacterial viability, SEM micrograph, and wettability properties of Ti-Fe-Cu, Zr-Fe-Al, and Zr-W-Cu thin films on the SS316L substrate, as compared with the corresponding SS316L bare substrate, are shown in Figure 6. The CLSM images (Figure 6a) show a population of viable bacteria on all samples after 2 h of incubation except for Zr-Fe-Al. However, after 4 h, the total population of bacteria on Ti-Fe-Cu, Zr-Fe-Al, and Zr-W-Cu appear dead. In contrast to the bare substrate, the total bacteria population appears viable. Moreover, Figure 6b shows a community of bacterial cells gathering more on the bare SS316L substrate, which means the bare substrate encourages bacterial metabolism, eventually leading to biofilm. The random and scarcely distributed bacterial cells on the nanotextured surfaces of the thin film metallic glass samples reveal their bactericidal properties. The SEM images in Figure 6b show large clusters of bacterial cells formed on the SS316L surface after 4 h of incubation time, in agreement with the RFU (Figure 6a).

The hydrophobic properties (Figure 6c) of the thin films create a strong affinity between the film and the bacterial surface, thereby enhancing the penetration of nanoparticles (due to nanoscale surface features) through the bacterial cell membrane after membrane damage by ROS [57,58] (discussed in Section 3.3: Bactericidal/antibacterial mechanism) and cause a high mortality rate. This is evidenced by randomly dispersed bacterial colonies/attachments after 4 h, as bacterial metabolic activities were deterred. The metallic glass-coated SS316L samples deterred bacteria colonisation and biofilm formation as opposed to the bare SS316L, which may favour conditions for biofilm development over longer durations.

#### 3.2.4. Live/Dead Quantitative Analysis

A valuable quantitative metric that provides insights into the dynamics of bacterial populations and their responses to the nanotextured metallic glass surface is the “active cell ratio”, which represents the fraction of bacterial cells within a population that is metabolically active or capable of growth/proliferation. The use of fluorescent dyes like SYTO 9 and propidium iodide (PI) to selectively stain live and dead bacterial cells unveils the active cell ratio, which is estimated using Equation (1) [59].

Figure 7 shows a graphical comparison of the active cell ratio of the films on (a) Ti and (b) SS316L substrates. The relatively similar variability bars represent standard deviation (*n* = 3 independent experiments); statistical significance is indicated where applicable in the active cell ratio results. The total population of bacteria attached to the bare substrates (Ti and SS316L) appear viable for the 2 and 4 h incubation time. However, a different trend is observed for the metallic glass-coated substrates, where there are relatively more dead than viable bacteria of the total attached bacteria with time. Overall, among the metallic glass samples, the rate of bacteria immobility is higher on Zr-Fe-Al, as more bacteria died after 2 h of incubation time on both substrates. These early effects are attributed to enhanced surface interaction and immobilisation associated with higher roughness (Table 3, especially on SS316L Ra = 8.1 nm), combined with wettability effects. At the same time, bactericidal activity is confirmed through viability assays rather than inferred from adhesion alone [60].

The active cell ratio of *P. aeruginosa* on bare and coated Ti and SS316L substrates after 2 h and 4 h of incubation is summarised in Table 4. Viability is expressed as a percentage relative to the corresponding bare-substrate control (100%), with the percentage reduction calculated accordingly. On Ti substrates, all coatings reduced bacterial viability relative to the bare surface. Ti–Fe–Cu exhibited a marked time-dependent response, with the active cell ratio decreasing from 70 ± 5% (2 h) to 10 ± 4% (4 h), corresponding to an increase in reduction from 30 ± 4% to 90 ± 5%. Zr–Fe–Al showed low viability at 2 h (25 ± 3%) with 75 ± 5% reduction, followed by a partial increase at 4 h (38 ± 6%, 62 ± 5% reduction). Zr–W–Cu maintained consistently low viability at both time points (18 ± 4% at 2 h; 20 ± 5% at 4 h), corresponding to reductions of 80 ± 5% and 78 ± 5%, respectively.

On SS316L substrates, a more pronounced bactericidal response was observed, particularly at 4 h. Ti–Fe–Cu reduced viability from 80 ± 6% (2 h) to 0 ± 1% (4 h), corresponding to 20 ± 2% and 100 ± 1% reduction, respectively. Similarly, Zr-Fe-Al decreased from 58 ± 3% (2 h) to 0 ± 1% (4 h) (52 ± 3% to 100 ± 1% reduction). Zr-W-Cu showed no reduction at 2 h (100 ± 1%) but reached 0 ± 1% viability (100 ± 1% reduction) at 4 h, indicating a delayed yet strong bactericidal effect.

Overall, the coatings induced a clear, time-dependent decrease in bacterial viability, with stronger effects observed on SS316L compared to Ti. The absence of detectable live-cell fluorescence and dominance of red signal at 4 h indicates a transition from metabolically active to membrane-compromised bacterial states. In several cases, particularly for coated SS316L surfaces at 4 h, the complete loss of detectable viable-cell signal within the analysed fields of view is consistent with strong bactericidal performance.

### 3.3. Bactericidal/Antibacterial Mechanism

**Figure 8:**
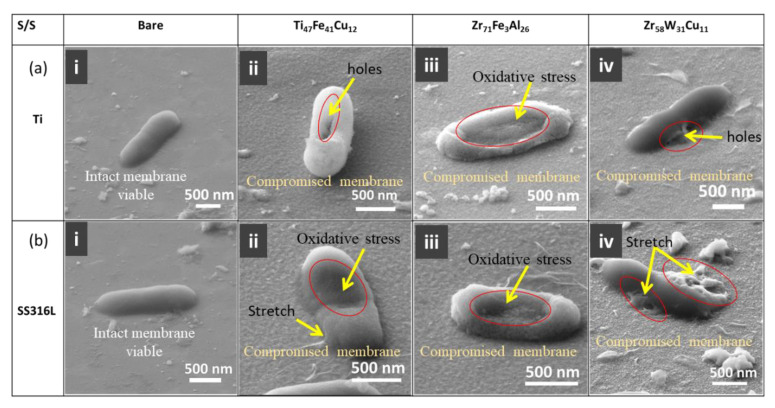
FIB-SEM images of viable and dead *P. aeruginosa* on the bare and coated substrates: (**a**) Ti and (**b**) SS316L. The red-circled areas indicate a compromised bacterial cell wall. (**i**) Viable cells with intact cell membranes and smooth morphology. (**ii**) Early-stage membrane damage characterised by the appearance of pores/holes and localised oxidative stress. (**iii**) Advanced membrane compromises under oxidative stress, showing surface depression and structural weakening. (**iv**) Severe membrane disruption with extensive perforations and deformation; stretching of the cell body is also evident. Scale bar: 500 nm.

Figure 8 shows FIB-SEM images of *P. aeruginosa* on the nanotextured thin film coatings and bare substrates. The observed antibacterial response arises from a combination of composition, nanoscale roughness (Ra), and wettability (WCA), which together influence bacterial adhesion and cell integrity [61,62]. As shown in Figure 8, the membrane of the rod-like bacterial cell on the Ti and SS316L substrates (Figure 8(ai,bi)) is progressively disrupted on the coated surfaces (Figure 8(aii–aiv,bii–biv)), with the red-encircled regions indicating compromised cell walls and loss of viability.

From a wettability (WCA) perspective, bacterial adhesion and viability exhibit time-dependent behaviour. As discussed in Section 3.1, *P. aeruginosa* adheres to both hydrophilic and hydrophobic surfaces, but through different mechanisms. Hydrophilic surfaces promote adhesion via hydrogen bonding and hydration layer interactions [63], whereas hydrophobic surfaces induce membrane stress due to their lipophilic nature, disrupting the lipid bilayer and leading to structural damage and cell death [64]. These effects are reflected in the progressive membrane degradation observed across the coated samples.

From a composition perspective, the coatings exhibit distinct antibacterial pathways. Ti-Fe-Cu and Zr-W-Cu contain Cu, which contributes to enhanced bactericidal activity through ion release and associated reactive species generation, whereas Zr-Fe-Al lacks Cu and instead relies more strongly on surface-mediated interactions. As reported in our previous study, these films form CuO (Ti-Fe-Cu), Fe_2_O_3_ (Zr-Fe-Al), and WO_3_/ZrO_2_ (Zr-W-Cu) in physiological-like environments [34], which is analogous to bacterial suspension conditions (LB broth containing Na^+^ and Cl^−^) that promote oxide formation [54,65]. These metal oxide nanoparticles, which are also a function of the nanoscale surface features, exhibit inhibitory effects on bacteria [66,67] through electrostatic interactions with negatively charged bacterial membranes [68], leading to localised reduction reactions and membrane damage [62,69], as highlighted in Figure 8(aii,aiv).

From a surface roughness (Ra) standpoint, nanoscale topography contributes primarily to bacterial immobilisation and mechanical interactions rather than serving as the sole bactericidal factor. Surfaces with higher Ra increase the number of contact points between the bacterial cell envelope and the coating, thereby promoting physical stress and facilitating subsequent chemical interactions. This is particularly relevant for Zr–Fe–Al, where higher roughness enhances early-stage cell attachment and immobilisation, thereby increasing susceptibility to membrane disruption observed in Figure 8.

In addition, ROS generated from metal oxide phases play a critical role in bactericidal activity. These highly reactive oxygen species (for example, Cu^2+^ and singlet oxygen ^1^O_2_ from Cu– Ti-containing coatings, respectively) induce oxidative stress, disrupt cellular metabolism, and damage membrane lipids and proteins [22,58,66,70,71], as evidenced by the extensive cell wall degradation in Figure 8(aiii,bii–biv). The mechanistic interpretation is also consistent with the supplementary electrochemical and surface characterisation data (Tables S1–S3 and Figures S3–S6), which indicate stable oxide-forming surfaces and nanoscale topographies capable of promoting membrane stress and oxidative interactions with bacterial cells.

Overall, the antibacterial performance of the coatings is best understood as a synergistic, multivariate effect, where composition governs ion release and ROS generation, surface roughness (Ra) controls mechanical interaction and bacterial immobilisation, and wettability (WCA) regulates adhesion dynamics and membrane stability.

Thus, the bactericidal efficacy of the films against *P. aeruginosa*, as shown in Figure 8, arises from the combined influence of metal oxide nanoparticle activity, surface nanotopography, and wettability-driven interactions, rather than any single dominant factor.

## 4. Conclusions

This study evaluated the antimicrobial properties and performance of nanostructured Ti_47_Fe_41_Cu_12_, Zr_71_Fe_3_Al_26_, and Zr_58_W_31_Cu_11_ polymorphic thin film metallic glasses on Ti and SS316L substrates against *P. aeruginosa*. These coatings, with a nanoscale roughness ranging 1.6–8.1 nm and a thickness of 190–298 nm, exhibit hydrophilic-to-hydrophobic surface properties that are critical for antimicrobial performance. Short-term assays (2–4 h) demonstrated reduced bacterial adhesion and viability relative to bare substrates, particularly with the Zr58W31Cu11 coating, which showed the highest efficiency. This significant reduction in viable cells (>60%) of the coatings is likely associated with a range of factors, including hydrophobicity, release of ions by the nanotextured metal oxides, and generation of ROS. While longer-time (≥24 h) bactericidal testing is important to fully assess biofilm inhibition and validate mechanistic interpretations, the results reported in this paper highlight the potential of polymorphic metallic glass coatings as durable and clinically promising antimicrobial surfaces for biomedical implants.

Overall, this work provides a foundational understanding of how compositionally distinct TFMG biocompatibility coatings influence early bacterial adhesion and viability and establishes a basis for the rational design of next-generation antimicrobial surfaces, while acknowledging that further validation is required before clinical translation.

## Figures and Tables

**Table 1:** Co-sputtering deposition parameters for Ti-Fe-Cu, Zr-Fe-Al, and Zr-W-Cu nanotextured thin film metallic glass coatings, showing elemental target powers and deposition time.

Sample	Deposition Power (Watts)			Time (min)
	Element 1	Element 2	Element 3	
Ti–Fe–Cu	Ti = 150	Fe = 50	Cu = 50	30
Zr–Fe–Al	Zr = 150	Fe = 50	Al = 50	30
Zr–W–Cu	Zr = 150	W = 50	Cu = 50	30

**Table 2:** Film compositions, thickness, and roughness (Ra) of the co-sputtered nanostructured polymorphous thin film metallic glass samples on SS316L and Ti substrates. The value of 0.047 nm corresponds to the instrumental uncertainty/resolution obtained from the calibration and profile-fitting procedure of the AFM surface characterisation system and was constant for all measurements reported in this table.

Sample	Film Thickness (nm)	Surface Roughness (nm)	
		SS316L	Ti
Bare substrate	-	1.6	4.0
Ti_47_Fe_41_Cu_12_	190	3.5	3.0
Zr_71_Fe_3_Al_26_	298	8.1	2.0
Zr_58_W_31_Cu_11_	280	4.9	1.5

**Table 3:** Mean water contact angle (WCA) and surface roughness of the bare substrates (SS316L and Ti) and the as-deposited thin film metallic glass samples. The value of 0.047 nm corresponds to the instrumental uncertainty/resolution obtained from the calibration and profile-fitting procedure of the AFM surface characterisation system and was constant for all measurements reported in this table.

Samples	Mean WCA (Degree)		Roughness (nm)	
	SS316L	Ti	SS316L	Ti
Bare	99.0 ± 1.7	93.1 ± 7.4	1.6	4.0
Ti_47_Fe_41_Cu_12_	106.5 ± 3.5	83.4 ± 4.3	3.5	3.0
Zr_71_Fe_3_Al_26_	78.6 ± 5.2	71.1 ± 5.6	8.1	2.0
Zr_58_W_31_Cu_11_	94.5 ± 8.1	73.8 ± 2.8	4.9	1.5

**Table 4:** Quantitative assessment of bacterial viability on bare and coated Ti and SS316L substrates after 2 h and 4 h of incubation. Active cell ratio (%) represents the proportion of live (membrane-intact) bacteria determined from live/dead fluorescence imaging, reported as mean ± standard deviation from at least three independent biological replicates, each averaged over multiple fields of view. Reduction (%) is calculated relative to the corresponding bare substrate at each time point.

Substrate	Sample	Active Cell Ratio (%) 2 h	Reduction (%) 2 h	Active Cell Ratio (%) 4 h	Reduction (%) 4 h
Ti	Bare Ti	100 ± 1	0	100 ± 1	0
	Ti-Fe-Cu	70 ± 5	30 ± 4	10 ± 4	90 ± 5
	Zr-Fe-Al	25 ± 3	75 ± 5	38 ± 6	62 ± 5
	Zr-W-Cu	18 ± 4	80 ± 5	20 ± 5	78 ± 5
SS316L	Bare SS316L	100 ± 0	0	100 ± 0	0
	Ti-Fe-Cu	80 ± 6	20 ± 2	0 ± 1	100 ± 0
	Zr-Fe-Al	58 ± 3	52 ± 3	0 ± 1	100 ± 1
	Zr-W-Cu	100 ± 1	0 ± 1	0 ± 1	100 ± 1

## Data Availability

The original contributions presented in this study are included in the article/Supplementary Materials. Further inquiries can be directed to the corresponding authors.

## Supplementary Materials

The following supporting information can be downloaded at https://www.mdpi.com/article/10.3390/biom16060759/s1. Table S1. Fifteen trial matrix combinations of Ti- and Zr-based metallic glass thin films on a glass substrate. Table S2. Film compositions, thickness, and roughness (Ra) of the co-sputtered nanostructured thin film metallic glass samples on SS316L and Ti-6Al-4V substrates. Note that the surface roughness of SS316L and polished Ti-6Al-4V bare substrates is 14 nm and 4 nm, respectively. Table S3. Young’s modulus (E), surface hardness (H), and film adhesive strength (FAS) of the bare and thin film metallic glass-coated SS316L and Ti substrates. Figure S1. XRD patterns with PDF crystal-phase positions and amorphous % of the as-sputtered (i,ii) Ti-Fe-Cu, (iii,iv) Zr-Fe-Al, and (v,vi) Zr-W-Cu polymorphous thin film metallic glasses deposited on SS316L and Ti substrates. AFM of Ti-Fe-Cu, Zr-Fe-Al, and Zr-W-Cu thin film metallic glass on (b) SS316L and (c) Ti substrates. The inset shows the roughness (Ra) values of each film. Figure S2. XPS wide spectra of the as-deposited samples and their high-resolution scan: (a) Zr 3d, (b) W 4f, (c) Cu 2p, (d) Fe 2p, (e) Al 2p, and (f) Ti 2p. Figure S3. (a) XPS etch profile analysis, (b) Raman shift, and (c) corresponding image of Raman analysis location for Ti_47_Fe_41_Cu_12_ nanotextured polymorphous thin film metallic glass. Figure S4. (a) XPS etch profile analysis, (b) Raman shift, and (c) corresponding image of Raman analysis location for Zr_71_Fe_3_Al_26_ nanotextured polymorphous thin film metallic glass. Figure S5. (a) XPS etch profile analysis, (b) Raman shift, and (c) corresponding image of Raman analysis location for Zr_58_W_31_Cu_11_ nanotextured polymorphous thin film metallic glass. Figure S6. Representative images of (a) live and dead fluorophore preliminary investigation of *P. aeruginosa* after 2 and 4 h of incubation time, (b) wettability of rough/unpolished stainless steel and the as-deposited films.

## Abbreviations

AFM	Atomic Force Microscopy
BE	Bactericidal Efficacy
CFU	Colony-Forming Units
CLSM	Confocal Laser Scanning Microscopy
DLVO	Derjaguin–Landau–Verwey–Overbeek
EPS	Extracellular Polymeric Substances
FE-SEM	Field Emission Scanning Electron Microscopy
FIB	Focused Ion Beam
FIB-SEM	Focused Ion Beam Scanning Electron Microscopy
FOV	Field of View
ISO	International Organisation for Standardisation
LB	Luria–Bertani (broth)
PBS	Phosphate-Buffered Saline
PI	Propidium Iodide
Ra	Arithmetic Mean Surface Roughness
RFU	Relative Fluorescence Units
ROS	Reactive Oxygen Species
SEM	Scanning Electron Microscopy
SS316L	Stainless Steel 316L
TFMG	Thin Film Metallic Glass
UV	Ultraviolet
WCA	Water Contact Angle
XPS	X-ray Photoelectron Spectroscopy
XRD	X-ray Diffraction
