# Supplementary material for: Antimicrobial Properties of Ti- and Zr-Based Nanotextured Thin Film Metallic Glasses Against Pseudomonas aeruginosa
Source: Biomolecules. 2026 May 22;16(6):759. doi: 10.3390/biom16060759 (PMC13297426; doi:10.3390/biom16060759)
Supplement: Supplementary file 1 [file biomolecules-16-00759-s001.zip › biomolecules-4255782-supplementary.pdf]

Antimicrobial Properties of Ti- and Zr-based Nanotextured Thin Film Metallic Glasses against Pseudomonas aeruginosa

C.R. Onyeagba[1,4,5], J.M. Harris[3], T.E. Egbo[6]*, C. Brown[1,4,5], H. Wang[2,5], T. Tesfamichael[1,4,5]*

[1]School of Mechanical, Medical and Process Engineering, Faculty of Engineering, Queensland University of Technology, Brisbane, 4000, QLD, Australia

[2]School of Chemistry and Physics, Faculty of Science, Queensland University of Technology, Brisbane, 4000, QLD, Australia

[3]School of Biomedical Science, Faculty of Health, Queensland University of Technology, Brisbane, 4000, QLD, Australia

[4]Centre for Biomedical Technologies, Queensland University of Technology, Brisbane, 4000, QLD, Australia

[5]Centre for Materials Science, Queensland University of Technology, Brisbane, 4000, QLD, Australia

[6]Walter Reed Army Institute of Research-Armed Forces Research Institute of Medical Sciences

[*]Corresponding authors: timothy.e.egbo.mil@health.mil and t.tesfamichael@qut.edu.au

Supplementary Document

S1. Deposition of Ti- and Zr-Based Nanostructured Polymorphous Thin Film Metallic Glasses

Ti- and Zr-based PTFMG films were deposited on SS316L stainless steel and polished Ti substrates using a KJ Lesker PVD 75 magnetron sputtering system equipped with four 50 mm targets. Before deposition, all substrates were rinsed with distilled water and air-dried. High-purity (99.95–99.99%) sputtering targets of Ti, Zr, Fe, Cu, W, Al, Mg, Ta, and Hf (Maideli Advanced Material Co., Ltd) were selected based on glass-forming criteria, specifically atomic size mismatch, crystal structure incompatibility, and biocompatibility [38]. Three-element alloy systems (Tables S1–S2) were designed following the cluster + glue atom co-sputtering model [39], [40], with Ti or Zr acting as the primary cluster element and the remaining elements serving as glue atoms. Accordingly, the cluster element was sputtered at a higher power.

Deposition was performed at 150 W for the cluster target and 50 W for each glue target, with a working pressure of 10 mTorr for 60 minutes. The substrates were rotated at 10 rpm to improve coating uniformity, positioned 150 mm from the targets, and kept at ambient temperature. After evacuating the chamber to less than 2.0×10^{-6} mTorr, high-purity argon was introduced. Each target was pre-sputtered for 5 minutes to remove surface contaminants before film growth began. SS316L and Ti substrates with different initial roughness values (14 nm and 4 nm, respectively) were used to achieve final surface textures suitable for bactericidal nanostructures, following the recommended roughness threshold (< 10 nm).

Table S1. Fifteen trial matrix combinations of Ti- and Zr-based metallic glass thin films on a glass substrate.

**Sample**	**Deposition power (Watts)**			**Time (min)**
	Element 1	Element 2	Element 3	
Ti-Fe-Al	Ti = 100	Fe = 100	Al = 50	60
Ti-Fe-Zr	Ti = 100	Fe = 100	Zr = 50	60
Ti-W-Mg	Ti = 100	W = 100	Mg = 50	60
Ti-Hf-Mg	Ti = 100	Hf = 100	Mg = 50	60
Ti-Fe-Cu	Ti = 150	Fe = 100	Cu = 50	60
Ti-W-Cu	Ti = 150	W = 100	Cu = 50	60
Ti-Al-Cu	Ti = 150	Al = 100	Al = 50	60
Ti-Fe-Ta	Ti = 150	Fe = 100	Ta = 50	60
Zr-Fe-Al	Zr = 100	Fe = 50	Al = 50	60
Zr-W-Cu	Zr = 150	W = 100	Cu = 50	60
Zr-Fe-Ta	Zr= 150	Fe = 100	Ta = 50	60
Zr-Al-Cu	Zr = 150	Al = 50	Cu = 50	30
Zr-Al-Mg	Zr = 150	Al = 100	Mg = 50	60
Zr-W-Mg	Zr = 150	W = 100	Mg = 50	60

Zr-Fe-Cu	Zr = 150	Fe = 100	Cu = 50	60

Based on polymorphous content (> 50% amorphous phase) and microstructural uniformity, three representative samples, Ti-Fe-Cu (Sample 3), Zr-Fe-Al (Sample 5), and Zr-W-Cu (Sample 7) were selected for further analysis (**Table S2**). Because film stoichiometry (**Figure S2**), thickness, and amorphous/crystalline ratios (**Figure S1**) depend directly on deposition parameters. The values reported in **Table S2** are not intended to represent optimised properties.

Table S2. Film compositions, thickness, and roughness (Ra) of the co-sputtered nanostructured thin film metallic glass samples on SS316L and Ti-6Al-4V substrates. Note that the surface roughness of SS316L and polished Ti-6Al-4V bare substrates is 14 nm and 4 nm, respectively.

**Sample ID**	**Film stoichiometry**	**Thickness (nm)**	**Roughness (nm) on each substrate**	
			**SS316L**	**Ti-6Al-4V**
3	$Ti_{47}Fe_{41}Cu_{12}$	190	7	3
5	$Zr_{71}Fe_{3}Al_{26}$	298	3	2
7	$Zr_{58}W_{31}Cu_{11}$	280	2	1.5

S2. Mechanical Properties and Microstructure

Error! Not a valid bookmark self-reference. shows Young's modulus (E), surface hardness (H), and film adhesive strength (FAS) of the Ti- and Zr-based thin film metallic glasses. Generally, the bare SS316L and Ti substrates have a higher Young's modulus [1], [2], than the thin film metallic glass, which is beneficial in limiting bone stress shielding [3].

Table S3. Young's modulus (E), surface hardness (H), and film adhesive strength (FAS) of the bare and thin film metallic glass-coated SS316L and Ti substrates.

**Sample**	**SS316L substrate**		**Ti substrate**		**FAS (µN)/(MPa)**	
	***E* (GPa)**	***H* (GPa)**	***E* (GPa)**	***H* (GPa)**	SS316L	Ti
Bare	210	1.7 – 2.2	120	3.3 – 3.7	-	-
$Ti_{47}Fe_{41}Cu_{12}$	74.7 ± 8.4	2.9 ± 1.0	75.0 ± 8.3	0.7 ± 0.8	1921	2629
$Zr_{71}Fe_{3}Al_{26}$	93.6 ± 10.4	4.3 ± 1.2	93.8 ± 5.1	3.4 ± 0.5	624	2978
$Zr_{58}W_{31}Cu_{11}$	90.8 ± 9.6	1.8 ± 0.9	89.6 ± 4.9	1.3 ± 0.4	2159	2273

Moreover, the thin film metallic glasses exhibit higher surface hardness (0.7 – 4.3 GPa) than bare SS316L (1.7 – 2.2 GPa) and Ti (3.3 – 3.7 GPa). This affirms that surface coating could improve the mechanical functionality of a material [68], [69]. Also, the *E* and *H* values of the as-deposited thin film metallic glasses are closely matched with other studies: $Ti_{40}Zr_{10}Cu_{36}Pd_{14}$, $Zr_{46}Cu_{46}Al_8$, $Zr_{50}Cu_{50}$, and $Zr_{42}Cu_{42}Al_7Y_5$ [3], [4], [5], [6]. Moreover, the adhesive strength on the Ti substrate is excellent and higher than on the SS316L substrate, and this can be attributed to the higher hardness and lower roughness of the substrate. These tribological properties offer great benefits as a sustainable surface coating for Ti and SS316L substrates concerning industrial and biomedical applications.

The XRD pattern of Ti-Fe-Cu (i, ii), Zr-Fe-Al (iii, iv), and Zr-W-Cu (v, vi) PTFMG on SS316L and Ti substrates is shown in **Figure S1a**. The XRD pattern of the Ti-based (Ti-Fe-Cu) PTFMG shows a broad hump with about 55% amorphous characteristics on both substrates. Fe and Ti were identified as the major elements of the crystal phase (crystallites) on both substrates, respectively. This may be a substrate effect since Fe and Ti are constituents of the crystalline substrates (SS316 and Ti), respectively. The percentage of amorphous/crystalline characteristics of the polymorphic metallic glass thin films was deduced from the XRD software DIFFRAC.EVA V6 using the following equations:

$$\%\text{Amorphous} = \frac{\text{Global area} - \text{Reduced area}}{\text{Global area}} \times 100 \quad (1)$$

$$\%\ \text{Crystallinity} = 100 - \%\text{Amorphous} \quad (2)$$

The Zr-based (Zr-Fe-Al and Zr-W-Cu) PTFMGs showed broad XRD spectra on SS316L and Ti substrates. Zr-Fe-Al (**Figure S1 iii, iv**) demonstrates polymorphic properties with amorphous (crystalline) phase proportions of 76% (24%) and 67% (33%) on SS316L and Ti substrates, respectively. Fe and Zr3Fe were identified as the primary contributors to the crystal phase (crystallites) on both substrates, respectively.

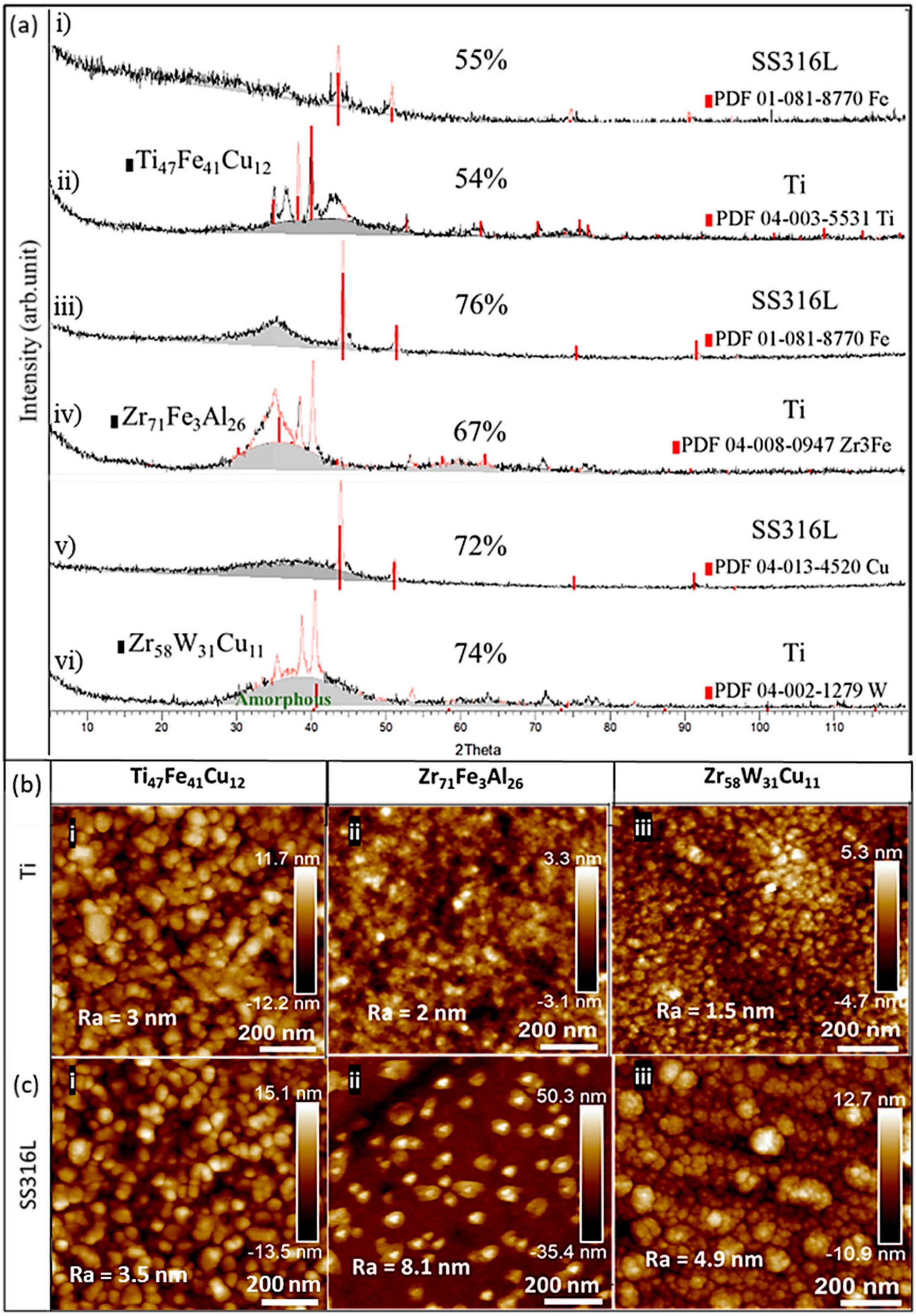

Figure S1. XRD patterns with PDF crystal phase positions and amorphous % of the as-sputtered; a (i, ii) Ti-Fe-Cu, (iii, iv) Zr-Fe-Al, and (v, vi) Zr-W-Cu polymorphous thin film metallic glasses on SS316L and Ti substrates. AFM of Ti-Fe-Cu, Zr-Fe-Al, and Zr-W-Cu thin film metallic glass on (b) SS316L and (c) Ti substrates. The inset shows the roughness (Ra) values of each film. Figure (a,b) is licensed under CC−BY 4.0 and reproduced from reference [7] © 2023 C.R. Onyeagba, M. Valashani, H. Wang, C. Brown, P. Yarlagadda, T. Tesfamichael. Published by Elsevier B.V.

The crystallite of Fe appears to be pronounced on the SS316L substrate, which may be a joint effect of the presence of Fe in the film ternary and SS316L, thus, substrate effect. The XRD of Zr-W-Cu in (**Figure S1 v, vi**) displays 72% (28%) and 74% (26%) of amorphous (crystalline) phase proportion on the SS316L and Ti substrates, respectively. Both Cu and W were identified as the crystallites on both substrates.

It is important to note that, in addition to the glass-forming ability, incompatibility of crystal structure (hexagonal close packing-HCP, body centred-BCC, face centred-FCC) and atomic size discourages vitrification and promotes the formation of a predominant amorphous phase [59]. In this study, the co-sputtered $Ti_{47}Fe_{41}Cu_{12}$, $Zr_{71}Fe_{3}Al_{26}$ and $Zr_{58}W_{31}Cu_{11}$ matrix featured a unique structure combination of HCP (Ti, Zr) - BCC (Fe, W), and FCC (Al, Cu). This combination also fosters high strength and stiffness (due to HCP and BCC) with good ductility (due to FCC) [60].

The AFM images of the as-deposited Ti-Fe-Cu, Zr-Fe-Al, and Zr-W-Cu thin film metallic glass are shown in **Figure S1 b and c, which** reveal relatively uniform nanostructures with the roughness of 1.5 nm - 3nm and 3.5 nm - 8.1 nm on Ti and SS316L substrate, respectively. These nanotextured surfaces have demonstrated good mechanical properties, as shown in **Table S3** and contributed to the bactericidal efficiency of the samples as discussed in section 3.3 of the main paper, by the generation of reactive oxygen species (ROS) substantiated in our previous study [8].

S3. XPS analysis of as-deposited samples

XPS wide scan (Figure S2) of the Ti- and Zr-based as-deposited samples shows that the samples contain C and O, which are surface contaminants introduced from the atmosphere, and no trace of these elements was observed after lightly etching the films. Thus, the stoichiometry and composition of the three films were obtained using the lightly etched XPS results.

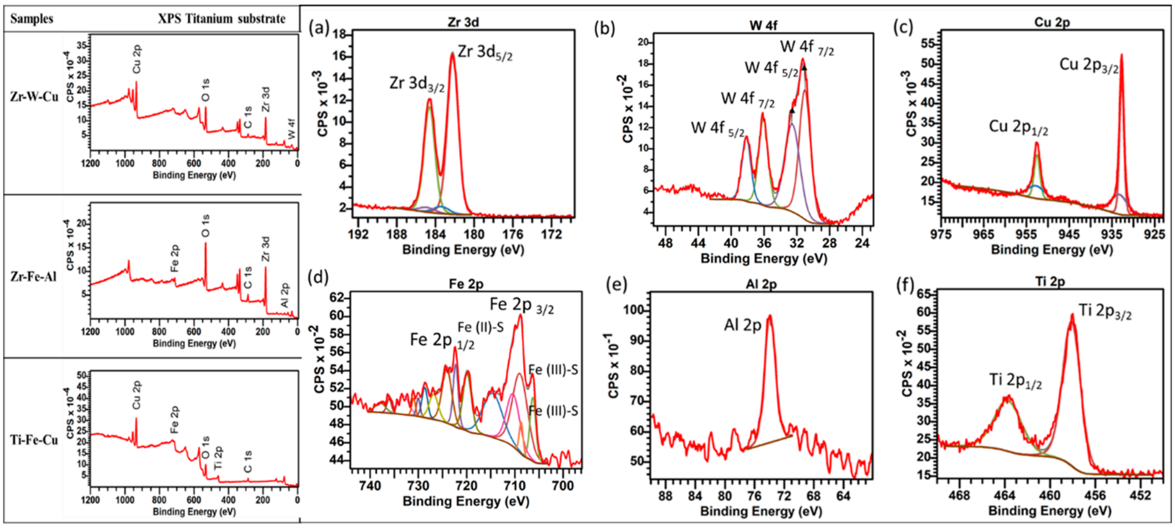

Figure S2 XPS wide spectra of the as-deposited samples and their high-resolution scan: (a) Zr 3d, (b) W 4f (c) Cu 2p (d) Fe 2p, (e) Al 2p, and (f) Ti 2p. Figure (a,b) is licensed under CC−BY 4.0 and reproduced from reference [7] © 2023 C.R. Onyeagba, M. Valashani, H. Wang, C. Brown, P. Yarlagadda, T. Tesfamichael. Published by Elsevier B.V.

The high-resolution XPS spectra (Figure S2) show Zr 3d core level (a) located at approximately 182.2 eV and 184.6 eV, which corresponds to ZrO_2 [45]. The W 4f spectrum was separated into two spin-orbit doublet peaks (b) corresponding BE of W oxidation states. While the W $4f_{5/2}$ and W $4f_{7/2}$ peaks located at BE of 37.8 and 35.6 eV are ascribed to the W^{6+} state, the W $4f_{5/2}$ and W $4f_{7/2}$ peaks at 33.1 and 31.6 eV are typical of the W metal, respectively. [9], [10], [11]. This further confirms the XRD indication of W as a crystallite and the formation of nanostructures. The Cu 2p core level (c) spectrum was fitted at 932.6 eV, which is assigned to Cu (0) [12]. The Fe 2p core level (d) was fitted at 710 eV from the main Fe 2p3/2 peak, which reflects Fe3+. The Al 2p core level spectrum (e) has a main peak located at 72.1 eV, denoting the presence of metallic aluminium. The Ti 2p core level spectrum (f) was fitted with two peaks with BEs of 458.5 eV and 464.2 eV, which correspond to Ti 2p3/2 and Ti 2p1/2, respectively, denoting Ti4+ [13], [14].

S.2.1 XPS and Raman Surface analysis of the samples in Simulated Body Fluid

XPS analysis **(Figure S3a, Figure S4a, Figure S5a)** and Raman analysis **(Figure S3b, Figure S4b, Figure S5b)** were carried out to determine the penetration of the Simulated Body Fluid (SBF) and understand the nature, surface chemistry and corrosion-resisting mechanism of the bare substrate and TFMG samples. Before measurement, the samples were thoroughly cleaned with deionized water and swiped with cotton to remove any reactants on the surface.

Figure S3a shows a change in the percentage of each polymorphous admixture constituting elements of the Ti-Fe-Cu sample with sputter/etching time. Carbon and oxygen were detected along with the constituent elements of the sample. C appears to be a surface contaminant, whereas the presence of oxygen after the etchings suggests the penetration of SBF deep into the bulk of the coating by the formation of oxides. The presence of oxygen oscillates in sync with the Fe and out of sync with Ti and Cu through the surface of the film, which suggests the presence of a layered oxide. Moreover, the presence of Ti provides stability for the passive film [15].

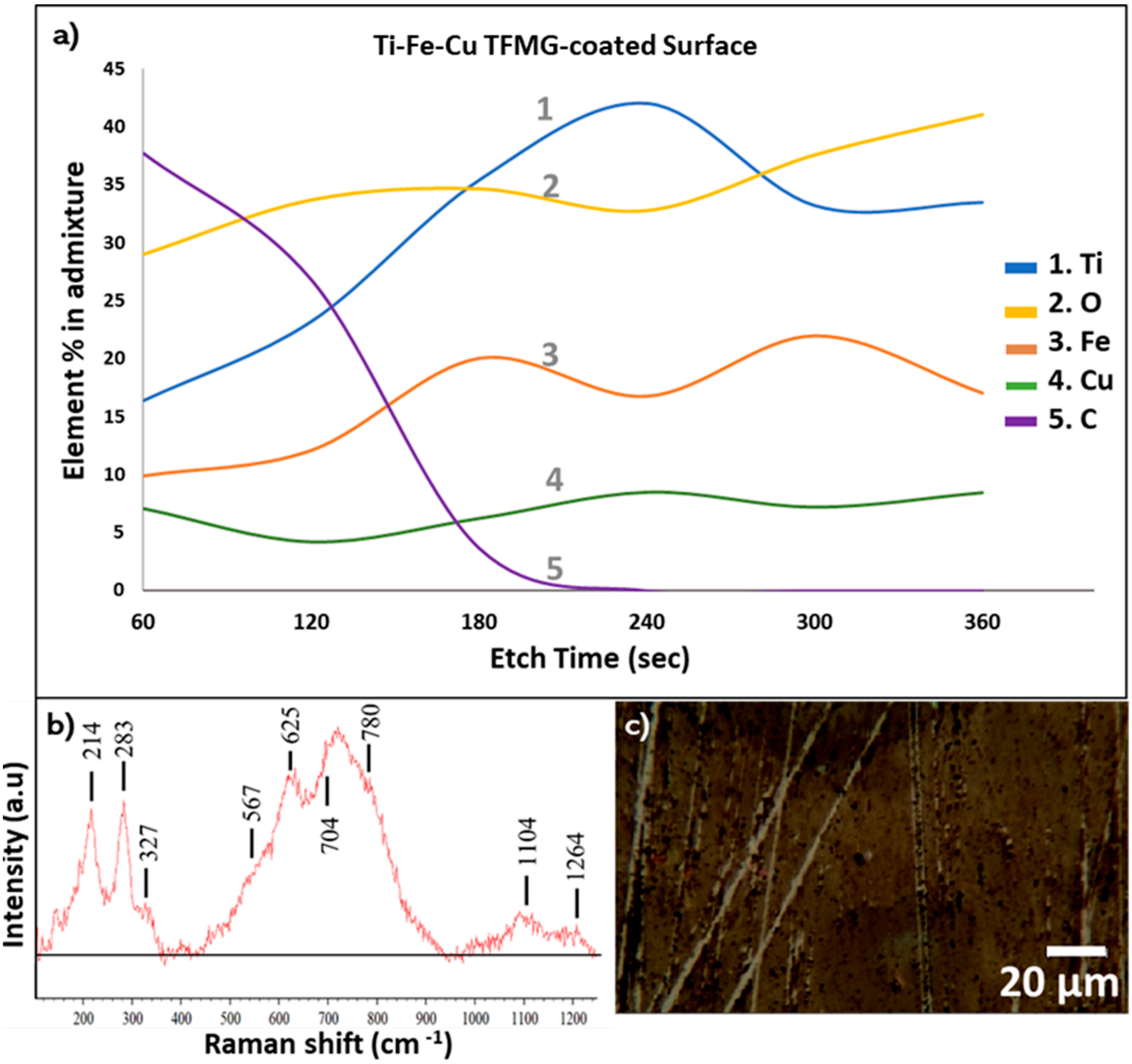

Figure S3. (a) XPS etch profile analysis, (b) Raman shift, and (c) corresponding image of Raman analysis location for $Ti_{47}Fe_{41}Cu_{12}$ nanotextured polymorphous thin film metallic glass. Figure is licensed under CC−BY 4.0 and reproduced from reference [8] © 2023 C.R. Onyeagba, G. Will, M. Barclay, C. Brown, H. Wang, T. Tesfamichael Published by Elsevier Ltd.

Furthermore, the Raman spectra (Figure S3b) show characteristic peaks that suggest a passive film of multiple metal oxides on the Ti-Fe-Cu sample. The strong sharp peaks at 214 cm^{-1} and 283 cm^{-1} are correlated with hematite Fe_2O_3, however, the expected weak peaks of hematite around 610 cm^{-1} and 662 cm^{-1} are not visible [16]. This may be due to the convolution of these peaks with the much stronger characteristic peak of Cu oxide at 625 cm^{-1}. The broad shoulder at 420 cm^{-1} to 567 cm^{-1} is correlated to an amorphous Cu-oxide phase [17], [18]. Since a single peak and a broad shoulder may not conclusively substantiate the presence of Cu oxide,

fundamental studies affirm that the primitive cell of Cu-oxide has very strong peaks at 298 cm^{-1} and 345 cm^{-1} [19]. Consequently, it can be concluded that the Ti-Fe-Cu thin film forms a protective oxide layer of predominantly Cu-oxide on the surface of the sample that reduces further. From Figure S4a and Figure S5a, carbon and oxygen were detected in addition to the expected constituent elements in Zr-Fe-Al and Zr-W-Cu samples, respectively. Again, C appears as a surface contaminant and disappears after slight etching, whereas the presence of oxygen suggests the formation of oxides into the bulk of the coatings and prevents further corrosion. These induced oxides for the Zr-Fe-Al and Zr-W-Cu samples were analysed by Raman as shown in Figure S4b and Figure S5b, respectively.

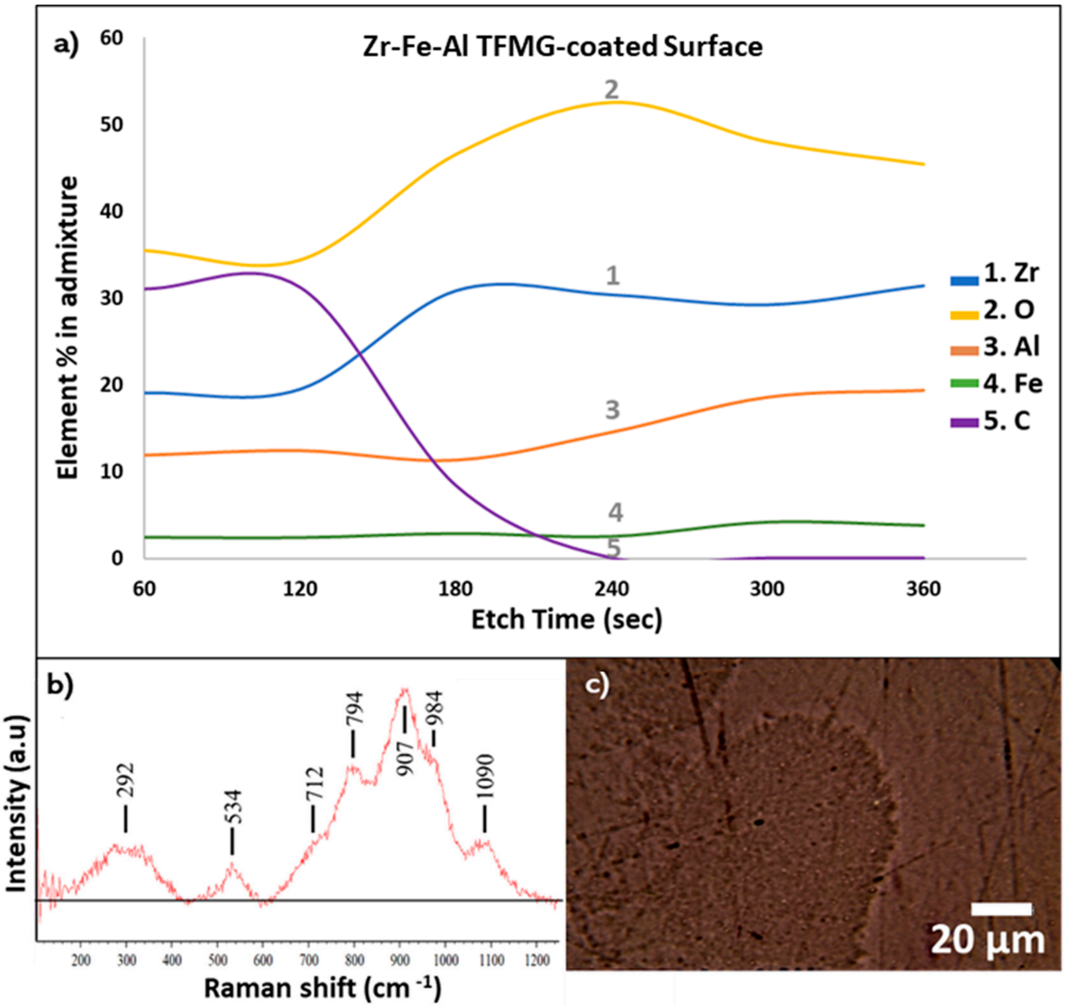

Figure S4. (a) XPS etch profile analysis, (b) Raman shift, and (c) corresponding image of Raman analysis location for $Zr_{71}Fe_3Al_{26}$ nanotextured polymorphous thin film metallic glass. Figure is licensed under CC−BY 4.0 and reproduced from reference [8] © 2023 C.R. Onyeagba, G. Will, M. Barclay, C. Brown, H. Wang, T. Tesfamichael Published by Elsevier Ltd.

The Raman spectra (Figure S4b) of the electrochemically tested Zr-Fe-Al sample show a weak peak at 1090 c,m^{-1} which is correlated with hematite Fe_2O_3. The broad peak at 292 cm^{-1} suggests a convolution of three weak peaks of hematite, magnetite and maghemite each made broader by the amorphous state of the sample. The presence of magnetite and maghemite is further substantiated by two peaks located at 534 cm^{-1} and 715 cm^{-1}, respectively [20], [21]. The strong peak at 907 cm^{-1} cannot be correlated with any material within the sample without introducing new structures such as silicate contamination and, thus, the formation of almandine $e_3Al_2(SiO_4)_3$ [22]. Moreover, almandine is the ferrous iron end member (transition zone) [23]. Therefore, the Zr-Fe-Al coated substrate forms a Fe-oxide layer of mixed phases (hematite, magnetite and maghemite) to resist corrosion.

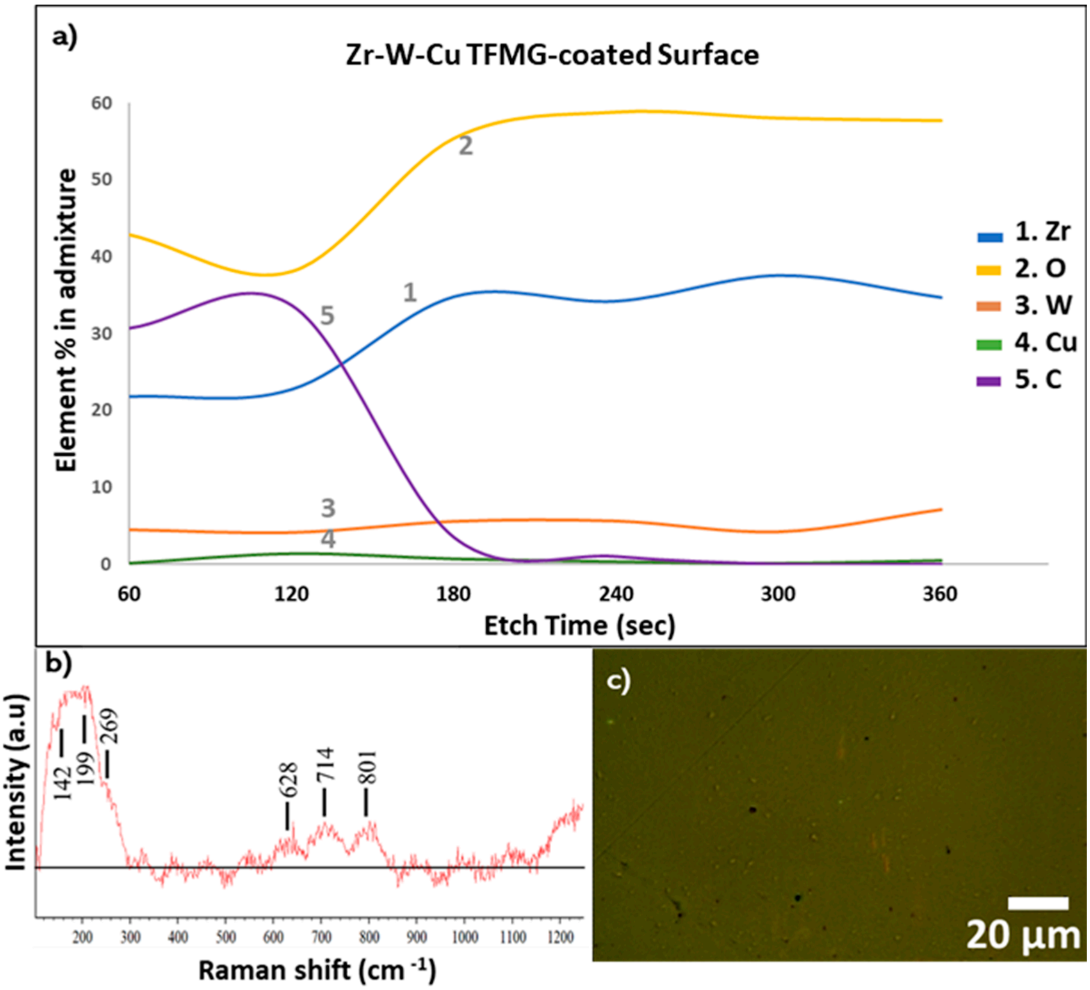

Figure S5. (a) XPS etch profile analysis, (b) Raman shift, and (c) corresponding image of Raman analysis location for $Zr_{58}W_{31}Cu_{11}$ nanotextured polymorphous thin film metallic glass. Figure is licensed under CC−BY 4.0 and

reproduced from reference [8] © 2023 C.R. Onyeagba, G. Will, M. Barclay, C. Brown, H. Wang, T. Tesfamichael Published by Elsevier Ltd.

The Raman spectrum (Figure S5b) of the electrochemical tested Zr-W-Cu sample shows a broad peak in the low frequency range of 140 cm^{-1} - 200 cm^{-1} with a shoulder centred at 269 cm^{-1}. The peak fitting analysis shows that this is better characterised by two convoluted peaks centred at 142 cm^{-1} and 199 cm^{-1}, respectively. These convoluted peaks correlate with monoclinic zirconium oxide (ZrO_2) [24] where the peaks are broad due to the amorphous nature of the sample, resulting in an overlap of the identified peaks. Some of the expected peaks (300 cm^{-1} - 400 cm^{-1}, 480 cm^{-1} and 650 cm^{-1}) of ZrO_2 are not present due to a blanketing effect from a much less Raman active tungsten oxide (WO_3) with small peaks at 714 cm^{-1} and 801 cm^{-1} [25]. Thus, the Zr-W-Cu coating on Ti substrate induces passive films of WO_3 and ZrO_2 to resist corrosion proliferation in the SBF solution. Overall, the Zr-W-Cu sample has oxide species from both the base (Zr) and supplementary (W) elements which became an additional factor for reducing corrosion proliferation significantly (nearly 450 times than the bare Ti-substrate as seen from the electrochemical testing) [8].

S3. Optimising surface roughness

To investigate the effect of roughness on SS316L, a preliminary experiment was conducted by depositing the films on a rougher SS316L substrate (Ra:14 nm). It was observed that the film/substrate adhesion weakened as the films delaminated from the substrates after 4 h incubation (Figure S6a), hence the loss of bactericidal efficacy. Figure S6a (i – ix) describes the state of the samples after 2 and 4 h incubation time. The as-deposited Ti-Fe-Cu (ii) observes total delamination from the substrate after 2 h as opposed to Zr-Fe-Al and Zr-W-Cu, where delamination was observed during and after 4h incubation time. Consequently, a similar trend (Figure S6a) of live (green) and attached bacteria is observed for the bare SS316L (i) and the Ti-Fe-Cu coated substrate. However, high bactericidal efficacy (Figure S6a) is evident for the

Zr-Fe-Al (iv) and Zr-W-Cu (v) coatings, as there are more dead bacteria (red) than live (green) until 4 h incubation (vii and ix) when delamination occurred. This weak film/substrate adhesion is a resultant effect of a rough substrate. As shown in Figure S6b, the bare SS316L is more hydrophobic than the samples and thus, more attached to bacteria. Moreover, an aggregation of live bacteria (biofilm) occurs on the bare substrate more than in the samples where a sparse distribution of bacterial populations is seen. This is due to the bactericidal efficacy of the samples, where bacterial metabolism is deterred. Overall, despite the good bactericidal efficacy of the as-deposited samples, rougher substrates deter their adhesion to the substrate, thus smooth substrates (Ra: 1.6 to 4 nm) are recommended for effective functionality.

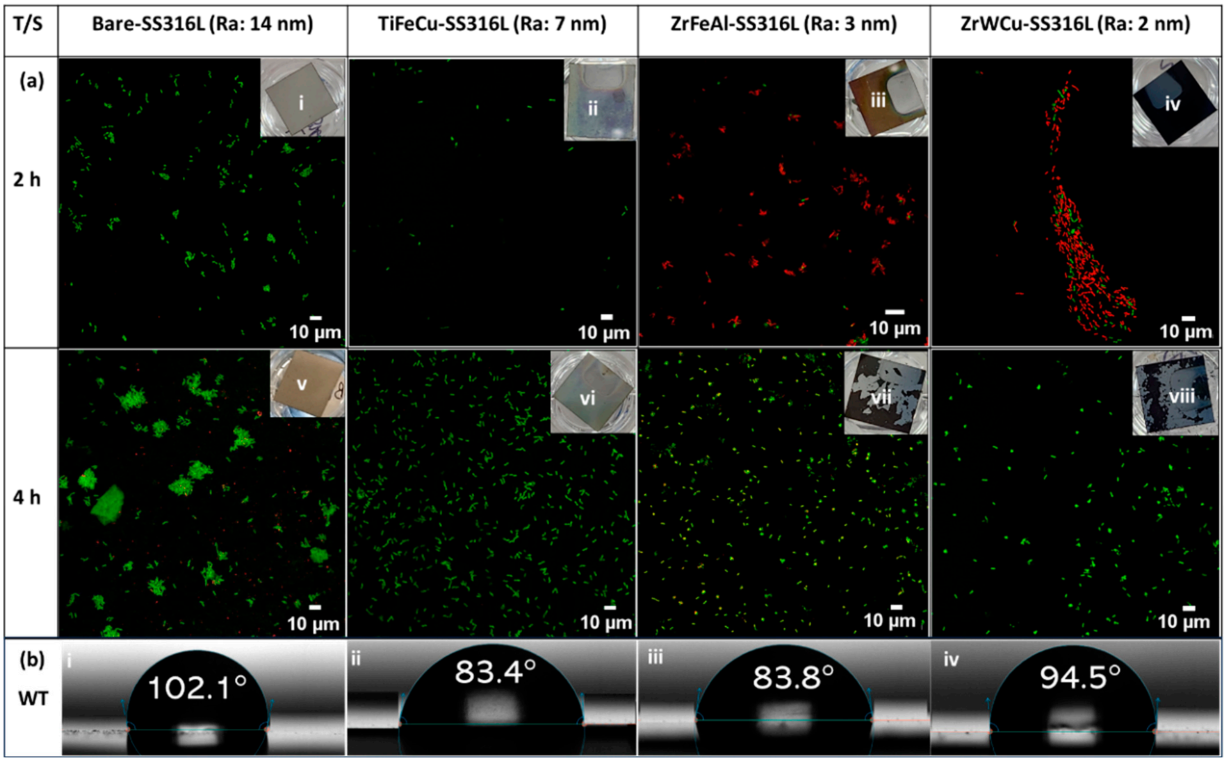

Figure S6. Representative images of (a) live and dead fluorophore preliminary investigation of P. aeruginosa after 2 and 4 h incubation time, (b) wettability of rough/unpolished stainless-steel and the as-deposited films.

Reference

[1] M. Rossi, F. Pierron, M. Štamborská, and F. Šimčák, "Identification of the Anisotropic Plastic Behaviour of Sheet Metals at Large Strains," in *Experimental and Applied Mechanics, Volume 4*, C. E. Ventura, W. C. Crone, and C. Furlong, Eds., in Conference Proceedings of the Society for Experimental Mechanics Series. , New York, NY: Springer New York, 2013, pp. 229–235. doi: 10.1007/978-1-4614-4226-4_27.

[2] "Titanium Properties," Kyocera SGS Europe. Accessed: June 03, 2023. [Online]. Available: https://kyocera-sgstool.co.uk/titanium-resources/titanium-information-everything-you-need-to-know/titanium-properties/

[3] A. Liens *et al.*, "On the Potential of Bulk Metallic Glasses for Dental Implantology: Case Study on Ti40Zr10Cu36Pd14," *Materials*, vol. 11, p. 249, Feb. 2018, doi: 10.3390/ma11020249.

[4] Juan Z., Guozheng K., and Wei R., "," 力学学报, vol. 52, no. 2, pp. 318–332, Apr. 2020, doi: 10.6052/0459-1879-20-038.

[5] M. Apreutesei *et al.*, "Microstructural, thermal and mechanical behavior of co-sputtered binary Zr–Cu thin film metallic glasses," *Thin Solid Films*, vol. 561, pp. 53–59, June 2014, doi: 10.1016/j.tsf.2013.05.177.

[6] G. J. Fan *et al.*, "Thermophysical and elastic properties of Cu50Zr50 and (Cu50Zr50)95Al5 bulk-metallic-glass-forming alloys," *Appl. Phys. Lett.*, vol. 89, no. 24, p. 241917, Dec. 2006, doi: 10.1063/1.2408634.

[7] C. R. Onyeagba, M. Valashani, H. Wang, C. Brown, P. Yarlagadda, and T. Tesfamichael, "Nanomechanical surface properties of co-sputtered thin film polymorphic metallic glasses based on Ti-Fe-Cu, Zr-Fe-Al, and Zr-W-Cu," *Surf. Interfaces*, vol. 40, p. 103090, Aug. 2023, doi: 10.1016/j.surfin.2023.103090.

[8] C. R. Onyeagba, G. Will, M. Barclay, C. Brown, H. Wang, and T. Tesfamichael, "Polymorphous nanostructured metallic glass coatings for corrosion protection of medical grade Ti substrate," *Intermetallics*, vol. 165, p. 108167, Feb. 2024, doi: 10.1016/j.intermet.2023.108167.

[9] Y. Tao *et al.*, "WO3–SiO2 nanomaterials synthesized using a novel template-free method in supercritical CO2 as heterogeneous catalysts for epoxidation with H2O2," *Mater. Today Chem.*, vol. 18, p. 100373, Dec. 2020, doi: 10.1016/j.mtchem.2020.100373.

[10] F. A. El Diwany, T. Al Najjar, N. K. Allam, and E. N. El Sawy, "Tungsten oxide/fullerene-based nanocomposites as electrocatalysts and parasitic reactions inhibitors for VO2+/VO2+ in mixed-acids," *Sci. Rep.*, vol. 12, no. 1, Art. no. 1, Aug. 2022, doi: 10.1038/s41598-022-18561-6.

[11] "Transition from Battery to Pseudocapacitor Behavior via Structural Water in Tungsten Oxide | Chemistry of Materials." Accessed: June 06, 2023. [Online]. Available: https://pubs.acs.org/doi/10.1021/acs.chemmater.6b05485

[12] M. C. Biesinger, L. W. M. Lau, A. R. Gerson, and R. St. C. Smart, "Resolving surface chemical states in XPS analysis of first row transition metals, oxides and hydroxides: Sc, Ti, V, Cu and Zn," *Appl. Surf. Sci.*, vol. 257, no. 3, pp. 887–898, Nov. 2010, doi: 10.1016/j.apsusc.2010.07.086.

[13] S. Komornicki, M. Radecka, and P. Sobaś, "Structural properties of TiO 2 –WO 3 thin films prepared by r.f. sputtering," *J. Mater. Sci.-Mater. Electron. - J MATER SCI-MATER ELECTRON*, vol. 15, pp. 527–531, Aug. 2004, doi: 10.1023/B:JMSE.0000032587.22033.d0.

[14] W. Xie, R. Li, and S. Q. Xu, "Enhanced photocatalytic activity of Se-doped TiO2 under visible light irradiation," *Sci. Rep.*, vol. 8, June 2018, doi: 10.1038/s41598-018-27135-4.

[15] Z. Liu *et al.*, "Novel low Cu content and Ni-free Zr-based bulk metallic glasses for biomedical applications," *J. Non-Cryst. Solids*, vol. 363, pp. 1–5, Mar. 2013, doi: 10.1016/j.jnoncrysol.2012.09.026.

[16] I. Chourpa *et al.*, "Molecular composition of iron oxide nanoparticles, precursors for magnetic drug targeting, as characterized by confocal Raman microspectroscopy," *The Analyst*, vol. 130, no. 10, pp. 1395–1403, Oct. 2005, doi: 10.1039/b419004a.

[17] V. S. Levitskii *et al.*, "Raman spectroscopy of copper oxide films deposited by reactive magnetron sputtering," *Tech. Phys. Lett.*, vol. 41, no. 11, pp. 1094–1096, Nov. 2015, doi: 10.1134/S106378501511022X.

[18] M. H. Chou, S. B. Liu, C. Y. Huang, S. Y. Wu, and C.-L. Cheng, "Confocal Raman spectroscopic mapping studies on a single CuO nanowire," *Appl. Surf. Sci.*, vol. 254, no. 23, pp. 7539–7543, Sept. 2008, doi: 10.1016/j.apsusc.2007.12.065.

[19] J. Chrzanowski and J. C. Irwin, "Raman scattering from cupric oxide," *Solid State Commun.*, vol. 70, no. 1, pp. 11–14, Apr. 1989, doi: 10.1016/0038-1098(89)90457-2.

[20] "Raman microspectroscopy of some iron oxides and oxyhydroxides - de Faria - 1997 - Journal of Raman Spectroscopy - Wiley Online Library." Accessed: May 26, 2023. [Online]. Available: https://analyticalsciencejournals.onlinelibrary.wiley.com/doi/abs/10.1002/%28SICI%291097-4555%28199711%2928%3A11%3C873%3A%3AAID-JRS177%3E3.0.CO%3B2-B

[21] M. Lübbe, A. M. Gigler, R. W. Stark, and W. Moritz, "Identification of iron oxide phases in thin films grown on Al2O3(0001) by Raman spectroscopy and X-ray diffraction," *Surf. Sci.*, vol. 604, no. 7, pp. 679–685, Apr. 2010, doi: 10.1016/j.susc.2010.01.015.

[22] "(17) (PDF) Raman Spectra of Silicate Garnets." Accessed: May 26, 2023. [Online]. Available: https://www.researchgate.net/publication/226662941_Raman_Spectra_of_Silicate_Garnets

[23] D. Zherebetskyy, S. Lebernegg, G. Amthauer, and M. Grodzicki, "Magnetic structure of almandine," *Phys. Chem. Miner.*, vol. 39, no. 5, pp. 351–361, May 2012, doi: 10.1007/s00269-012-0494-z.

[24] C. M. Efaw *et al.*, "Characterization of zirconium oxides part I: Raman mapping and spectral feature analysis," *Nucl. Mater. Energy*, vol. 21, p. 100707, Dec. 2019, doi: 10.1016/j.nme.2019.100707.

[25] S. Xie *et al.*, "Molybdenum Oxide/Tungsten Oxide Nano-heterojunction with Improved Surface-Enhanced Raman Scattering Performance," *ACS Appl. Mater. Interfaces*, vol. 13, no. 28, pp. 33345–33353, July 2021, doi: 10.1021/acsami.1c03848.
